# Synergy by Perturbing the Gram-Negative Outer Membrane: Opening the Door for Gram-Positive Specific Antibiotics

**DOI:** 10.1021/acsinfecdis.2c00193

**Published:** 2022-08-10

**Authors:** Charlotte M. J. Wesseling, Nathaniel I. Martin

**Affiliations:** Biological Chemistry Group, Institute of Biology Leiden, Leiden University, 2333 BE Leiden, The Netherlands

**Keywords:** Gram-negative bacteria, Gram-positive antibiotics, synergy, outer membrane, permeabilization, potentiators

## Abstract

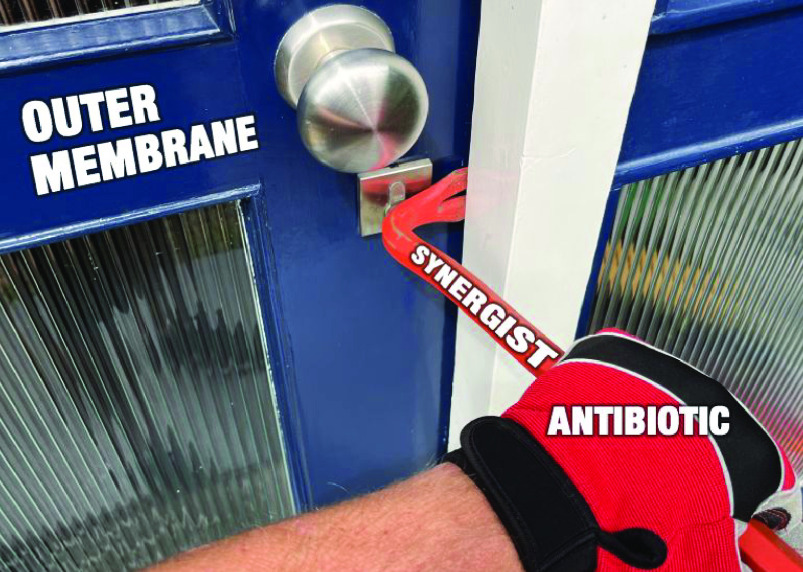

New approaches to target antibacterial agents toward Gram-negative bacteria are key, given the rise of antibiotic resistance. Since the discovery of polymyxin B nonapeptide as a potent Gram-negative outer membrane (OM)-permeabilizing synergist in the early 1980s, a vast amount of literature on such synergists has been published. This Review addresses a range of peptide-based and small organic compounds that disrupt the OM to elicit a synergistic effect with antibiotics that are otherwise inactive toward Gram-negative bacteria, with synergy defined as a fractional inhibitory concentration index (FICI) of <0.5. Another requirement for the inclusion of the synergists here covered is their potentiation of a specific set of clinically used antibiotics: erythromycin, rifampicin, novobiocin, or vancomycin. In addition, we have focused on those synergists with reported activity against Gram-negative members of the ESKAPE family of pathogens namely, *Escherichia coli*, *Pseudomonas aeruginosa*, *Klebsiella pneumoniae*, and/or *Acinetobacter baumannii*. In cases where the FICI values were not directly reported in the primary literature but could be calculated from the published data, we have done so, allowing for more direct comparison of potency with other synergists. We also address the hemolytic activity of the various OM-disrupting synergists reported in the literature, an effect that is often downplayed but is of key importance in assessing the selectivity of such compounds for Gram-negative bacteria.

The increasing occurrence of antibiotic resistance among Gram-negative pathogens highlights the need for novel antibacterial agents and therapeutic strategies. It is well established that Gram-negative bacteria are inherently harder to kill with antibiotics than Gram-positives, given the presence of the Gram-negative outer membrane (OM) as well as efflux pumps.^1−4^ Given the limited number of clinically effective anti-Gram-negative agents, there is an urgent need for new treatments against Gram-negative pathogens.^5−7^ This troubling reality is further exacerbated by increasing accounts of emerging resistance mechanisms against Gram-negative antibiotics, including extended spectrum β-lactamases (ESBLs) that can render even fifth-generation cephalosporins and carbapenems inactive,^8−11^ enzymes that structurally modify and deactivate aminoglycosides,^12−15^ and *mcr*-mediated polymyxin resistance.^16−27^ In this context, the World Health Organization (WHO) recently listed *Acinetobacter baumannii* (carbapenem-resistant), *Pseudomonas aeruginosa* (carbapenem-resistant), and the *Enterobacteriaceae* (carbapenem-resistant and ESBL-producing strains) as the bacterial pathogens of highest priority for the development of new antibiotics.^28^

The Gram-negative OM functions as a barrier that prevents many antibiotics, that are otherwise active against Gram-positive species, from reaching their targets.^3,29^ The OM itself consists of an asymmetrical lipid bilayer (see Figure 1A).^30^ The inner leaflet consist mostly of phospholipids and is similar to the cytoplasmic membrane.^31^ The outer leaflet is made up of an organized and fortified structure of densely packed lipopolysaccharides (LPSs) and Mg^2+^/Ca^2+^ cations that bridge the negatively charged phosphate groups of the lipid A component of LPS (see Figure 1B).^3,32^ Furthermore, the tightly packed saturated acyl chains result in a low level of membrane fluidity that limits the diffusion of hydrophobic compounds across the OM.^2,3^ The OM also contains porins, which function as size exclusion channels across the OM that mediate the diffusion of small hydrophilic molecules between the periplasm and the extracellular environment while keeping large, hydrophobic molecules, including many antibiotics, out.^1,2,29^ Additionally, when lipophilic or amphiphilic antibiotics do manage to cross the OM, multi-drug efflux pumps can transport these molecules back out.^1−3,29^ In many cases, the overexpression of efflux pumps provides an effective means for a Gram-negative pathogen to decrease its susceptibility to antibiotics.^3,33^ Taken together, their diverse resistance mechanisms and unique cellular features provide Gram-negative bacteria with a formidable range of defenses against antibacterial agents.

**Figure 1:**
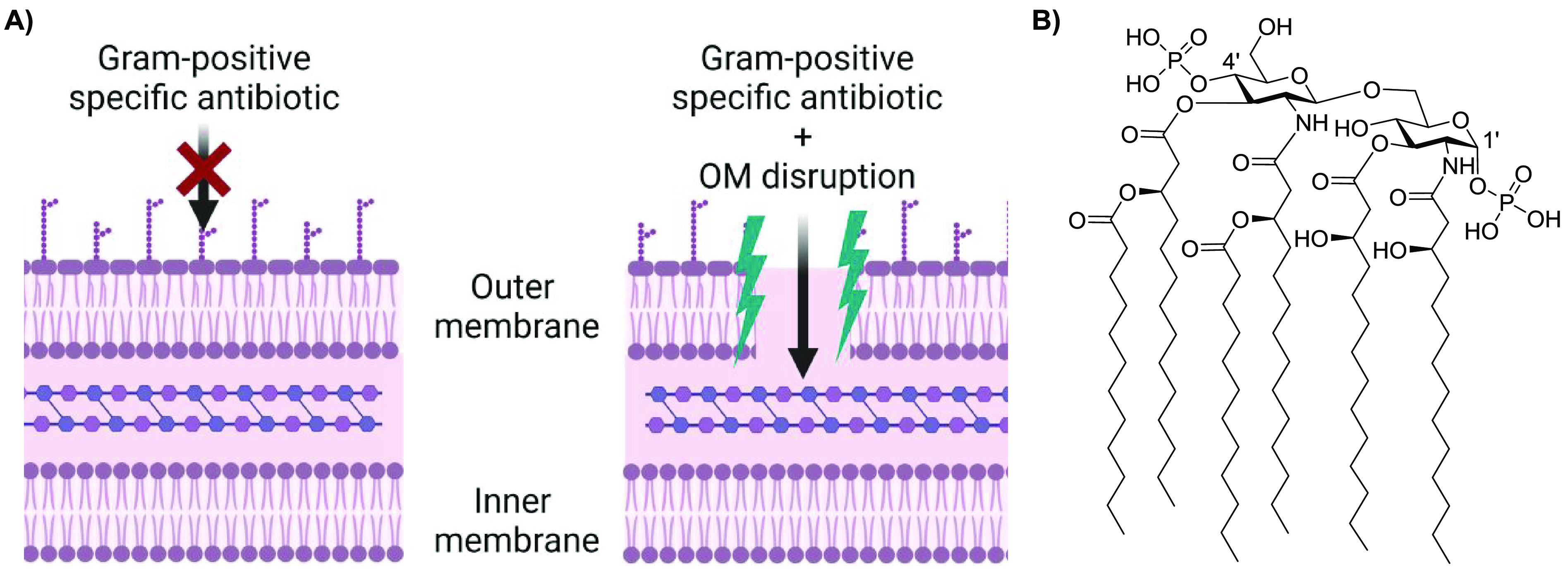
(A) Schematic depiction of the OM disruption required for potentiation of Gram-positive specific antibiotics (created with BioRender.com). (B) Lipid A (from *Escherichia coli* K-12), the hydrophobic anchor of LPS.

To address the specific challenges posed by Gram-negative bacteria, a number of new and innovative approaches are currently under investigation. Such strategies include interfering with LPS biosynthesis,^34−37^ targeting OM proteins such as the β-barrel assembly machine (BAM) complex,^34,38,39^ developing siderophore–antibiotic conjugates as Trojan horse agents, including the recently approved cefiderecol,^40−42^ co-administering different antibiotics to restrict or reverse antibiotic resistance,^43,44^ and blocking efflux pumps.^45−48^ In addition to these promising strategies, the development of agents that can selectively disrupt the OM offers the possibility of sensitizing Gram-negative bacteria to antibiotics that otherwise function only against Gram-positive bacteria.^3,7,32^ The pursuit of such synergists continues to be a very active field of research and is the basis for this Review.

The best-studied example of an OM-disrupting synergist is polymyxin B nonapeptide (PMBN), which is obtained by enzymatic degradation of the clinically used lipopeptide polymyxin B (PMB).^7,32^ The potentiating effects of PMBN were first reported in the 1980s, and in the decades since, a growing number of OM-disrupting synergists have been discovered.^7,32,49^ To date, a number of reviews have been published on the general topic of antibiotic synergy,^50−57^ including compounds that potentiate Gram-positive antibiotics through interactions with the OM^58^ and OM-disrupting synergists.^32,59−63^ However, a comprehensive overview of OM-disrupting synergists that also provides the reader with a direct comparison of both the potency and selectively of these compounds has, to date, been lacking. In this regard, the most widely accepted benchmark for synergistic activity is the so-called fractional inhibitory concentration index (FICI, Box 1).^64^ In this Review, we discuss only those synergists for which FICI values are reported or could be calculated from published data. The other criterion we have also chosen to emphasize is the selectivity of OM disruption associated with these synergists. In this regard, we pay special attention to the hemolytic activity reported for the various OM disrupters as a means of assessing their membrane specificity.

Box 1 **An important formalism in the field of synergy is the fractional inhibitory concentration index (FICI)**

The FICI is calculated from experimental minimum inhibitory concentration (MIC) data as shown in eq 1. A synergistic combination is generally defined as an FICI < 0.5. Additionally, it allows for a straightforward comparison of the potency of the synergistic combinations: the lower the FICI, the more potent the combination.
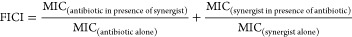
1

Among the Gram-negative bacteria for which OM-disrupting synergists have been reported, we have selected those pathogens noted on the WHO’s priority list: *A. baumannii*, *Escherichia coli*, *Klebsiella pneumoniae*, or *P. aeruginosa*.^28^ As for Gram-positive specific antibiotics whose activity is potentiated by OM-disrupting synergists, we have chosen to focus on clinically used agents that are most commonly evaluated for synergy with OM disrupters: erythromycin, rifampicin, vancomycin, and novobiocin.^7,58^ This criterion has, for example, led to the exclusion of OM-disrupting agents for which synergy was reported with macrolide antibiotics other than erythromycin.^65−68^ Also, while the specific media conditions used in antibacterial assays can strongly influence the outcome of synergy studies, for the sake of brevity, we do not include this level of detail here and instead provide clear referencing of the original studies wherein such information can be found. In addition, to further streamline the Review, synergists for which an OM-disrupting mechanism was not clearly demonstrated are not here discussed in detail.^69−77^ Furthermore, synergists that specifically engage with Gram-negative targets and subsequently cause OM disruption as a secondary effect are not discussed in this Review.^78−86^

The scope of the synergists included in this Review ranges from peptides to synthetic small molecules and small polymers of <1500 Da. In this regard, protein-based OM disrupters such as the membrane attack complex (MAC),^87^ lactoferrin,^88^ and the bactericidal/permeability-increasing protein (BPI)^89^ or larger polymers or polymer-like agents^90−97^ will not be discussed. This Review is further organized on the basis of the chemical families of the synergists covered. We begin with cyclic peptides based on PMBN, followed by linear peptides, cationic steroids, peptide–steroid hybrids, and small molecules. For each subgroup of synergists, a summary table has been assembled to provide a convenient comparative overview of FICI values. These tables also include the identity of the Gram-negative species and companion antibiotics employed in generating the FICIs. In addition, where possible, we have included the reported hemolytic activity of each synergist to provide an indication of its selectivity for Gram-negative cells.

## 1 Peptide-Based Potentiators

### 1.1 Polymyxin-Derived Synergists

**Figure 2:**
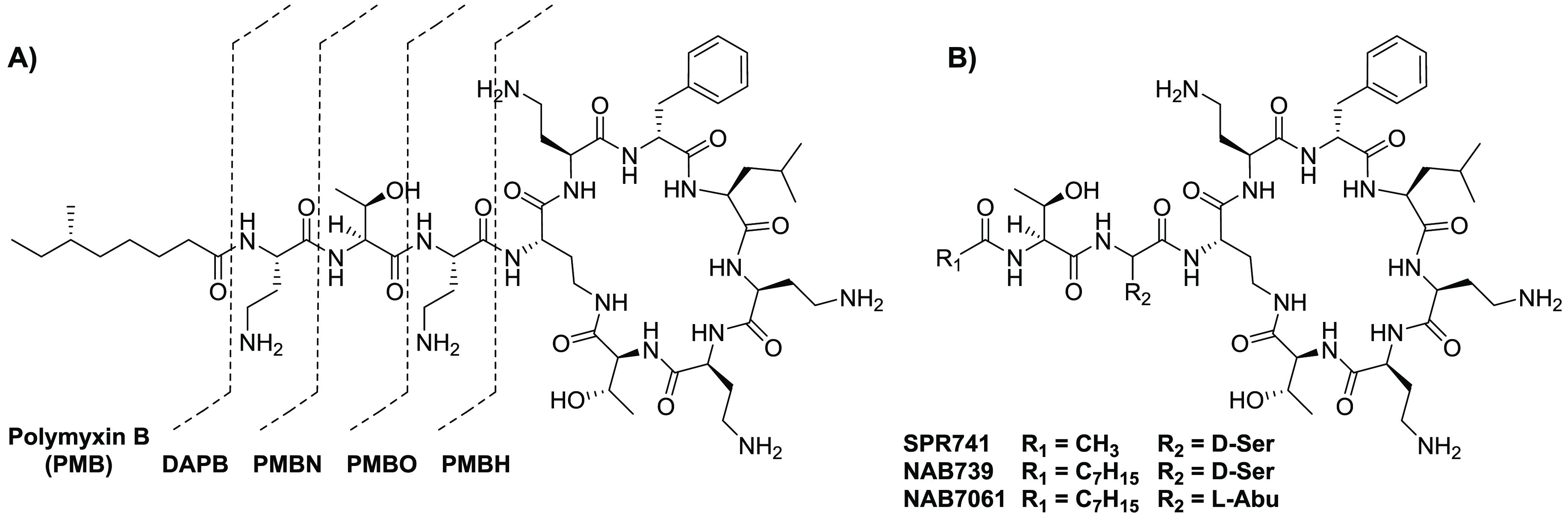
Molecular structures of (A) polymyxin B (PMB), deacylpolymyxin B (DAPB), polymyxin B nonapeptide (PMBN), polymyxin B octapeptide (PMBO), and polymyxin B heptapeptide (PMBH) and (B) PMBN analogues SPR741, NAB739, and NAB7061.

Polymyxin-derived synergists have been extensively reviewed in the past, and therefore only a concise summary of these analogues is here included.^7,32,63^ PMBN is derived from the parent lipopeptide PMB (see Figure 2A). Unlike its parent compound, PMBN has no inherent antimicrobial activity, nor is it nephrotoxic.^7,98^ In their landmark 1983 paper, Martti and Timo Vaara demonstrated that the combination of PMBN with hydrophobic, generally Gram-positive-specific, antibiotics results in a potent synergistic effect (see Table 1).^32,49^ In this regard, PMBN is often used as a benchmark for synergistic activity.^7^ Apart from PMBN, other truncated derivatives of PMB, like deacylpolymyxin B (DAPB), polymyxin B octapeptide (PMBO), and polymyxin B heptapeptide (PMBH), also display synergistic activity (Figure 1A and Table 1).^32^ The peptide macrocycle is of key importance for these synergists, as linear PMBN variants lose their synergistic activity.^99^

**Table 1:** Synergistic Activity of Polymyxin Analogues

name	ref	FICI^a^	pathogen	antibiotic
PMBN	(105)	0.013^a^	*E. coli*	rifampicin
PMBO	(105)	0.013^a^	*E. coli*	rifampicin
PMBH	(105)	0.020^a^	*E. coli*	rifampicin
DAPB	(105)	0.043^a^	*E. coli*	rifampicin
SPR741	(106)	0.06	*E. coli*	rifampicin
NAB739	(100)	0.126	*A. baumannii*	rifampicin
NAB7061	(100)	0.055	*E. coli*	rifampicin

a FICI calculated using eq 1 from MIC values reported in the cited reference.

A new generation of PMBN analogues containing only three positive charges was developed more recently.^100,101^ SPR741, previously named NAB741, has passed the Phase I clinical trials (see Figure 2B).^7^ Like PMBN, SPR741 has no lipophilic tail, resulting in improved renal clearance compared to PMB and other analogues that have a lipophilic tail, such as NAB739 and NAB7061.^101^ NAB7061 has little inherent antimicrobial activity but is a very potent synergist, while NAB739 has very potent antimicrobial activity (Table 1).^102^ Remarkably, this difference in activity between NAB739 and NAB7061 is attributed to the absence of one hydroxyl group in NAB7061 (see Figure 2B).^100^ NAB739 has been reported to exhibit generally moderate synergistic activity against wild-type strains, with the exception of the *A. baumannii* strain indicated in Table 1.^100,103^ Interestingly, against *mcr*-positive strains, the loss of antimicrobial activity for NAB739 is accompanied by a significant increase in its synergistic activity, an effect also noted for colistin.^103,104^

### 1.2 Dilipidated Polymyxins

**Figure 3:**
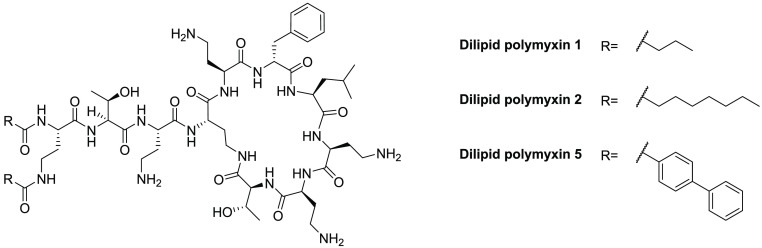
Molecular structures of the dilipidated polymyxin analogues.

Polymyxin analogues bearing additional lipid tails have also been explored to test the hypothesis that additional hydrophobicity might enhance membrane interactions.^107^ To generate these variants, a variety of acyl tails were added to both amino groups of the N-terminal 2,4-diaminobutyric acid (Dab) residue of PMB (Figure 3).^107,108^ The introduction of simple propyl lipids, as in analogue **1**, led to a complete loss of inherent activity (MIC ≥ 64 μg/mL), while the analogues **2** and **5**, bearing larger, more hydrophobic groups, maintained moderate activity, with MICs of 4–64 μg/mL against most Gram-negative bacteria.^107^ Notably, the reduced inherent activity was accompanied by a higher synergistic potential (Table 2), indicating that these dilipidated analogues have an increased capacity to disrupt the OM.^107^ Also of note is the reported activity of analogues **2** and **5** against Gram-positive bacteria (MICs of 8–32 μg/mL) compared to colistin, which has no such activity (MIC > 128 μg/mL).^107^

**Table 2:** Synergistic Activities of Dilipidated Polymyxin Analogues

name	ref	FICI	pathogen	antibiotic	hemolytic activity^a^
dilipid polymyxin 1	(107)	0.02	*P. aeruginosa*	rifampicin	<10% (1 h)
dilipid polymyxin 2	(107)	0.26	*P. aeruginosa*	novobiocin	<10% (1 h)
dilipid polymyxin 5	(107)	0.31	*P. aeruginosa*	rifampicin	<10% (1 h)

a Non-hemolytic is defined as <10% hemolysis compared to positive control, with incubation times denoted in parentheses.

### 1.3 Linear Peptide-Based Synergists

In most reviews published on the topic of OM-targeting synergists, relatively little attention has been paid to linear peptides. Peptides have several drawbacks, including poor metabolic stability, low bioavailability, potential immunogenicity, and high production costs.^109−111^ To improve their metabolic stability, the structures of peptides can be adapted by a number of approaches, including peptidomimetics, lipidation, head-to-tail cyclization, N- and C-terminus modifications, backbone stereochemistry changes, and incorporation of unnatural amino acids.^109,110,112−116^ Improvements to the bioavailability of peptides have also been explored by applying formulation techniques, adjusting the properties of peptides, or linking them to a moiety to improve passage over the blood–brain barrier.^109−111^ These advances, combined with the development of more economical methods for peptide synthesis, support a future role for peptide-based therapeutics, with a number of antimicrobial peptides (AMPs) already in (pre)clinical development.^117−121^

An increasing number of peptide synergists that function through OM disruption have been reported in the literature (see Table 3). In some studies, panels of structurally similar peptides are screened, resulting in the identification of multiple hits with FICI <0.5. In such cases, we have opted to select up to four of the most potent synergists to limit the number of peptides. Given that most peptide-based synergists are derived from specific lead proteins or AMPs, we have divided the linear peptide synergists accordingly, both in the discussion below and in the overview in Table 3.

**Table 3:** Overview of Linear Peptide-Based Synergists

name^a^	ref	peptide sequence^b^	FICI	pathogen	antibiotic	hemolytic activity^c^
**Cathelicidin-Derived Peptides**						
FK16	(130)	FKRIVQRIKDFLRNLV	0.25	*P. aeruginosa*	vancomycin	<10% (1 h)
KR-12-a2	(131, 214)	KRIVQRIKKWLR-NH_2_	0.156	*P. aeruginosa*	erythromycin	<10% (1 h)
L-11	(132)	RIVQRIKKWLR-NH_2_	0.070	*A. baumannii*	vancomycin	NR
D-11	(132, 133)	rivqrikkwlr-NH_2_	0.032	*A. baumannii*	rifampicin	<10% (1 h)
novicidin	(134)	KNLRRIIRKGIHIIKKYF	0.018	*E. coli*	rifampicin	<10% (1 h)
G2	(135)	RGARIVVIRVAR-NH_2_	0.38	*P. aeruginosa*	erythromycin	NR
R2	(135)	RRARIVVIRVAR-NH_2_	0.27	*P. aeruginosa*	erythromycin	NR
DP7	(138, 215)	VQWRIRVAVIRK	0.25	*P. aeruginosa*	vancomycin	<10% (1 h)
indopt 10	(135)	ILKWKIFKWKWFR-NH_2_	0.38	*P. aeruginosa*	erythromycin	NR
CLS001	(138, 140)	ILRWPWWPWRRK-NH_2_	0.28	*P. aeruginosa*	vancomycin	10% (30 min)

**Lactoferrin-Derived Peptides**						
P10	(141)	FWQRNIRKVKKK-NH_2_	0.113	*P. aeruginosa*	novobiocin	<10% (1 h)
P14	(141)	FWQRNIRKVKKKI-NH_2_	0.113	*P. aeruginosa*	novobiocin	<10% (1 h)
P22	(141)	RFWQRNIRKYRR-NH_2_	0.431	*P. aeruginosa*	novobiocin	<10% (1 h)
P2–16	(142)	FWRNIRIWRR-NH_2_	0.116	*P. aeruginosa*	novobiocin	NR
P12	(145, 216)	RRWQWRMKKLGA	0.43	*E. coli*	erythromycin	<10% (2 h)
P15	(145)	FK-Bip-RRWQWRMKKLGA^d^	0.38	*E. coli*	erythromycin	NR
P18	(145)	PAWFKARRWAWRMLKKAA	0.38	*E. coli*	erythromycin	NR

**Thrombin-Derived Peptides**						
peptide 6	(148)	VFRLKKWIQKVI-NH_2_	0.094	*E. coli*	rifampicin	<10% (20 h)
peptide 14	(148)	VFRLKKAIQKVI-NH_2_	0.078	*E. coli*	erythromycin	<10% (20 h)
peptide 19	(148)	VFRLKKWIQKVA-NH_2_	0.078	*E. coli*	rifampicin	<10% (20 h)

**Histatin-Derived Peptides**						
Nal-P-113	(153, 155)	Ac-AKR-Nal-Nal-GYKRKF-Nal-NH_2_^e^	0.38	*E. coli*	vancomycin	>10% (1 h)
Bip-P-113	(153, 155)	Ac-AKR-Bip-Bip-GYKRKF-Bip-NH_2_^d^	0.38	*E. coli*	vancomycin	>10% (1 h)

**Other Natural AMPs, Their Hybrids, and Derivatives**						
buforin II	(156, 217)	TRSSRAGLQFPVGRVHRLLRK	0.312	*A. baumannii*	rifampicin	<10% (1 h)
esculentin 1b	(157, 218)	GIFSKLAGKKLKNLLISG-NH_2_	0.36	*E. coli*	erythromycin	>10% (1 h)
HE2α	(158, 162)	VHISHREARGPSFRICVGFLGPRWARGCSTGN	0.3	*E.coli*	rifampicin	<10% (1 h)
HE2β2	(158, 162)	GDVPPGIRNTICRMQQGICRLFFCHSGTGQQHRQRCG	0.2	*E.coli*	rifampicin	<10% (1 h)
anoplin	(159)	GLLKRIKTLL	0.3125	*P. aeruginosa*	rifampicin	<10% (1 h)
magainin II	(160, 217)	GIGKFLHAAKKFAKAFVAEIMNS-NH_2_	0.312	*P. aeruginosa*	rifampicin	>10% (1 h)
cecropin A	(160, 165)	KWKLFKKIEKVGQNIRDGIIKAGPAVAVVGQATQIAK-NH_2_	0.312	*P. aeruginosa*	rifampicin	<10% (1 h)
CAME	(219, 220)	KWKLFKKIGIGAVLKVLTTG-NH_2_	0.375	*A. baumannii*	erythromycin	<10% (1 h)
CAMA	(219, 220)	KWKLFKKIGIGKFLHSAKKF-NH_2_	0.25	*A. baumannii*	erythromycin	<10% (1 h)
HPMA	(219, 221)	AKKVFKRLGIGKFLHSAKKF-NH_2_	0.313	*A. baumannii*	erythromcyin	<10% (1 h)^n^
H-TriA_1_	(168, 169)	v-dab-GswS-Dab-dab-FEV-alle-A^f^^,^^g^	0.002	*E. coli*	rifampicin	<10% (30 min)^n^
SLAP-S25	(173)	Ac-Dab-I-Dab-I-Dab-fL-Dab-vLA-NH_2_^f^	0.031	*E. coli*	rifampicin	<10% (1 h)
A13	(159)	GWWKRIKTWW	0.375	*K. pneumoniae*	rifampicin	<10% (1 h)
A17	(159)	KWWKRWKKWW	0.3125	*P. aeruginosa*	rifampicin	>10% (1 h)
A21	(159)	KWWKKWKKWW	0.3125	*K. pneumoniae*	rifampicin	<10% (1 h)
L7A	(139)	LNLKALAAVAKKIL-NH_2_	0.31	*E. coli*	rifampicin	<10% (1 h)
S1	(181, 184)	Ac-KKWRKWLAKK-NH_2_	0.38	*A. baumannii*	vancomycin	<10% (1 h)^n^
S1-Nal	(181, 184)	Ac-KKWRKWLAKK-Nal-NH_2_^e^	0.27	*A. baumannii*	vancomycin	<10% (1 h)^n^
S1-Nal-Nal	(181, 184)	Ac-KKWRKWLAKK-Nal-Nal-NH_2_^e^	0.27	*A. baumannii*	vancomycin	>10% (1 h)

**Peptide Synergists via Library Screening**						
peptide 79	(180, 185)	KKWRKWLKWLAKK-NH_2_	0.14	*E. coli*	rifampicin	<10% (1 h)
peptide 1	(71, 222)	KLWKKWKKWLK-NH_2_	0.02	*K. pneumoniae*	rifampicin	<10% (1 h)
peptide 2	(71, 188)	GKWKKILGKLIR-NH_2_	0.04	*K. pneumoniae*	rifampicin	<10% (1 h)
peptide D1	(71)	klwkkwkkwlk-NH_2_	≤0.03	*K. pneumoniae*	rifampicin	NR
peptide D2	(71)	gkwkkilgklir-NH_2_	≤0.04	*K. pneumoniae*	rifampicin	NR

**Peptide Synergists from Phage Display**						
EC5	(131, 186)	RLLFRKIRRLKR	0.266	*P. aeruginosa*	erythromycin	<10% (24 h)

**Designed Peptides**						
peptide 4	(187)	KFFKFFKFF	0.03	*E. coli*	rifampicin	>10% (30 min)
peptide 5	(187)	IKFLKFLKFL	0.06	*E. coli*	rifampicin	NR
peptide 7	(187)	CKFKFKFKFC	0.20	*E. coli*	rifampicin	NR
ΔFm	(191)	Ac-GΔFRKΔFHKΔFWA-NH_2_^h^	0.3	*E. coli*	rifampicin	<10% (1 h)
ΔFmscr	(191)	Ac-GΔFRKΔFKAΔFWH-NH_2_^h^	0.14	*E. coli*	rifampicin	<10% (1 h)
LK-L8P	(223)	Ac-LKKLLKLPKKLLKL-NH_2_	0.18	*E. coli*	erythromycin	<10% (4 h)
LK-L11P	(223)	Ac-LKKLLKLLKKPLKL-NH_2_	0.47	*E. coli*	erythromycin	<10% (4 h)
KL-L6P	(223)	Ac-LKKLLPLLKKLLKL-NH_2_	0.33	*E. coli*	erythromycin	>10% (4 h)
KL-L9P	(223)	Ac-LKKLLKLLPKLLKL-NH_2_	0.12	*E. coli*	erythromycin	<10% (4 h)
zp12	(196)	GIKRGIIKIIKRIKRI-NH_2_	0.25	*K. pneumoniae*	vancomycin	NR
zp16	(196)	GIKRGIIKIIRRIKRI-NH_2_	0.06	*K. pneumoniae*	vancomycin	<10% (1 h)
K4	(197, 198)	WRKWRKWRKWRK-NH_2_	0.2	*K. pneumoniae*	rifampicin	<10% (1 h)
K5	(197, 198)	WRKWRKWRKWRKWRK-NH_2_	0.2	*E. coli*	rifampicin	<10% (1 h)

**Lipopeptide Synergists**						
paenipeptin 1	(199, 200)	C_6_-Dab-I-Dab-fL-Dab-vLS-NH_2_^f^^,^^i^	0.125^o^	*E. coli*	rifampicin	<10% (30 min)
paenipeptin 9	(199)	C_8_-Dab-I-Dab-fL-Dab-vL-Dab-NH_2_^f^^,^^j^	≤0.03^o^	*K. pneumoniae*	rifampicin	<10% (30 min)
paenipeptin 15	(199)	Cbz-Dab-I-Dab-fL-Dab-vLS-NH_2_^f^^,^^k^	≤0.03^o^	*K. pneumoniae*	rifampicin	<10% (30 min)
paenipeptin 16	(199)	Cha-Dab-I-Dab-fL-Dab-vLS-NH_2_^f^^,^^l^	0.06^o^	*K. pneumoniae*	rifampicin	<10% (30 min)
dUSCL 2	(201)	C_10_-K(C_10_)KKK-NH_2_^m^ (Figure 4A)	0.07	*P. aeruginosa*	rifampicin	<10% (1 h)
dUSCL 6	(201)	C_10_-K(C_10_)KGK-NH_2_^m^ (Figure 4A)	0.25	*P. aeruginosa*	rifampicin	<10% (1 h)
UTBLP 5	(202)	C_8_-K(C_8_)KKKK-NH_2_^j^ (Figure 4B)	≥0.016	*P. aeruginosa*	novobiocin	NR
UTBLP 6	(202)	C_8_-K(C_8_)K(Me)K(Me)K(Me)K(Me)-NH_2_^j^ (Figure 4B)	0.047	*A. baumannii*	rifampicin	NR

**Lipopeptidomimetic Synergists**						
dUSTBP 2	(206)	Figure 4C	≥0.250	*P. aeruginosa*	rifampicin	<10% (1 h)
dUSTBP 5	(206)	Figure 4C	≥0.125	*P. aeruginosa*	rifampicin	<10% (1 h)
dUSTBP 8	(206)	Figure 4C	≥0.002	*A. baumannii*	novobiocin	<10% (1 h)
OAK C_12_(_ω7_)	(212)	Figure 4D	≤0.073^o^	*E. coli*	rifampicin	>10% (3 h)
OAK C_12_	(212)	Figure 4D	≤0.211^o^	*E. coli*	rifampicin	>10% (3 h)
OAK C_10_	(212)	Figure 4D	≤0.036^o^	*E. coli*	rifampicin	<10% (3 h)^n^
OAK C_8_	(212)	Figure 4D	≤0.078^o^	*E. coli*	rifampicin	<10% (3 h)^n^
OAK C_14(ω5)_OOc_10_O	(213)	Figure 4D	0.20^o^	*K. pneumoniae*	rifampicin	<10% (3 h)^n^

a Compound names are provided as given in the cited literature references.

b Lowercase letters indicate d-amino acids.

c Non-hemolytic is defined as <10% hemolysis compared to positive control, with incubation times denoted in parentheses; NR denotes no data reported.

d Bip = biphenylalanine.

e Nal = β-naphthylalanine.

f Dab = 2,4-diaminobutyric acid.

g alle = d-*allo*-isoleucine.

h ΔF = α,β-didehydrophenylalanine.

i C_6_ = hexanoyl.

j C_8_ = octanoyl.

k Cbz = benzyloxycarbonyl.

l Cha = cyclohexylalanyl.

m C_10_ = decanoyl.

n Concentration tested was lower than 100 μg/mL.

o FICI calculated from MIC values reported in the cited literature references.

#### 1.3.1 Cathelicidin Antimicrobial Peptides

The cathelicidins are AMPs that play an important role in the innate immune defense system of mammals and function by binding to bacterial membranes, resulting in their destabilization and lysis.^122−125^ In addition to their direct antibacterial activity, cathelicidins have also been found to play a role in recruiting immune cells to the site of infection as well as in LPS neutralization.^56,122,126^ The sole human cathelicidin-AMP gene encodes for hCAP-18, which is cleaved by proteases into the active LL-37.^123−125^ The mature LL-37 peptide forms an amphipathic α-helix that, upon interaction with bacterial cell surfaces, is associated with a detergent-like antimicrobial activity.^127−129^ Recently, a truncated version of LL-37, termed FK16, was reported to potentiate the activity of vancomycin against *P. aeruginosa* (Table 3).^130^ Similarly, the Kuipers group showed that another LL-37-derived sequence, termed KR-12-2, is able to synergize with azithromycin (and erythromycin, Table 3).^131^ Further optimization of the peptide sequence resulted in peptide L11, which was also synthesized as the d-amino acid variant (D11) as a means of improving serum stability (Table 3).^131,132^ These peptides were screened in combination with multiple antibiotics against different Gram-negative strains, and OM disruption assays verified their mode of action.^131−133^

In addition to the human cathelicidins, derivatives of cathelicidins from other mammals have also been screened for synergistic activity, including novicidin (sheep), bactenectin (bovine), and indolicidine (bovine).^122,134,135^ Among these, only novicidin was reported to display potent synergy (Table 3).^134^ In the case of bactenectin, which normally contains a disulfide bridge, a number of linear analogues have been prepared, including peptides G2, R2, and DP7, which were found to exhibit OM disruption and moderate synergy (Table 3).^135−138^ In the case of indolicidin, structure–activity relationship (SAR) studies have led to the discovery of the synergists Indopt 10 and CLS001 (Table 3). CLS001 is particularly effective and displays synergy with both vancomycin and azithromycin against multiple Gram-negative pathogens.^135,138^ Marketed under the name Omiganan, CLS001 is also much less hemolytic than indolicidin and is currently in clinical trials for the treatment of skin-related infections.^102,139,140^

#### 1.3.2 Lactoferrin-Derived Peptides

Lactoferrin is a multifunctional protein found in mammals and plays key roles in the human immune system. Lactoferrin has inherent activity against a range of bacterial, fungal, and viral pathogens, and in the case of Gram-negative bacteria, it can disrupt the OM.^88^ Based on the LPS-binding region of lactoferrin, known as LF11, the Martínez-de-Tejada group synthesized a series of LF11 homologues (Table 3) that were screened in combination with novobiocin for synergistic activity.^141^ Based on these findings, a new generation of peptide synergists was designed using PEptide DEscriptors from Sequence (PEDES) software to predict OM-permeabilizing sequences.^142^ The peptides thus obtained (i.e., peptide P2-16, Table 3) generally showed synergistic activity on par with that of the original series.^142^ Given the abundance of lactoferrins in other mammals, Svendsen and co-workers also investigated a series of peptides derived from bovine lactoferrin for both antimicrobial activity and synergistic activity.^143−146^ This led to the identification of a 12-mer peptide termed P12, along with P15, a 15-mer containing biphenylalanine (Bip), and a longer 18-mer termed P18, all of which were found to exhibit moderate synergy with erythromycin when tested against *E. coli* (Table 3).

#### 1.3.3 Thrombin-Derived Peptides

Thrombin is an enzyme that plays a critical role in coagulation, and recent studies have also shown that certain thrombin-derived C-terminal peptides are capable of binding to LPS and neutralizing its toxic and inflammatory effects.^147^ Given the capacity of PMB to also bind and neutralize LPS, our group was interested in assessing whether these thrombin-derived peptides might also exhibit the synergistic behavior of PMBN. To this end, we prepared a series of 12-mer thrombin-derived peptides and showed that a number of them are, indeed, potent synergists.^148^ The most active synergist thus identified (peptide **6**, Table 3) was further investigated by means of an alanine scan, leading to the discovery of more potent variants (peptides **14** and **19**, Table 3). Notably, these peptides were found to be non-hemolytic, and their synergistic activity was shown to extend to rifampicin, erythromycin, and novobiocin against multiple Gram-negative strains, including those with *mcr*-mediated resistance.^148^

#### 1.3.4 Histatins

The histatins are a unique group of histidine-rich peptides found in human saliva that play roles in defending against infection as well as in aiding wound-healing.^149^ Among the most common histatins, the 24 amino acid histatin **5** has been shown to bind Lipid A and has endotoxin-neutralizing properties.^150^ SAR studies with histatin **5** led to the identification of a 12-mer sub-region termed P-113 that exhibits antimicrobial activity against Gram-positive and Gram-negative bacteria.^149,151−153^ Further structural optimization to enhance the stability of P-113 led to analogues incorporating β-naphthylalanine (Nal) and Bip residues to yield Nal-P-113 and Bip-P-113 and wherein the 4th, 5th, and 12th histidine resides were replaced by Nal or Bip, respectively (Table 3).^153^ Bip-P-113 and Nal-P-113 exhibit antimicrobial activity and improved serum proteolytic stability, and they were also found to permeabilize LPSs containing large unilamellar vesicles used to model the Gram-negative OM.^153,154^ These findings prompted investigation of vancomycin potentiation by Bip-P-113 and Nal-P-113, revealing both to exhibit moderate synergy.^155^ However, a notable drawback of Bip-P-113 and Nal-P-113 is their significantly increased hemolytic activity relative to that of P-113.^153^

#### 1.3.5 Other Natural AMPs, Their Hybrids, and Derivatives

A number of other naturally occurring AMPs have been reported to potentiate antibiotics that are otherwise excluded by the OM. These AMPs are all polycationic and include buforrin II, esculentin 1b, sphistin, HE2α, HE2β2, anoplin, magainin II, and cecropin A (Table 3).^156−160^ The sources of these AMPs are diverse and include toads, wasp venom, or even the human male reproductive tract.^158,159,161^ The AMPs here discussed have all been reported to disrupt the OM,^157,159,162−164^ bind to LPS, and/or show endotoxin-neutralizing activity.^156,160,165,166^ In general, these AMPs exhibit modest FICIs (0.2–0.36), which has also led to interest in hybrids and derivatives with enhanced synergistic activity. For example, Park and co-workers developed a series of hybrid peptide synergists, termed CAME, CAMA, and HPMA, containing sequences derived from crecopin A, magainin II, and melittin (Table 3).^165,167^ Other approaches include truncation, as in the case of the lipopeptide AMPs tridecaptin A_1_ and B_1_ (TriA_1_ and TriB_1_), which themselves exhibit potent inherent anti-Gram-negative activity and, when truncated, were found to be effective synergists.^168−171^ Specifically, removal of the TriA_1_ N-terminal lipid yielded H-TriA_1_, which was found to be much less active as an antibiotic but exhibited very potent synergism when combined with rifampicin, resulting in an FICI of 0.002 against *E. coli* (Table 3).^168,169^ Like the tridecaptins, the recently discovered paenipeptins contain a number of Dab residues and have been the subject of SAR studies.^172^ These efforts led to the discovery of a potent paenipeptin-inspired synergist termed SLAP-S25, which effectively potentiates the activity of rifampicin and vancomycin against *E. coli* (Table 3).^173^ In addition to OM disruption, the binding of SLAP-S25 to LPS and phosphatidylglycerol (PG) was established, suggesting that SLAP-S25 is also an inner membrane disrupter.^173^ This was confirmed by dose-dependent uptake of propidium iodide and release of cellular contents in cells treated with SLAP-S25.^173^ Notably, SLAP-S25 was also demonstrated to effectively enhance the *in vivo* activity of colistin against a colistin-resistant strain of *E. coli* in both *Galleria mellonella* and mouse infection models.^173^

Originally isolated from wasp venom, anoplin is one of the smallest known amphipathic, α-helical AMPs.^159,161^ Multiple SAR investigations have been performed to improve its antimicrobial activity and stability.^174−178^ A recent study with anoplin reported the systematic introduction of tryptophan and lysine residues to determine the optimal hydrophobicity, amphipathicity, and number of positive charges required for antibacterial activity and minimal cytotoxicity.^159^ A number of these analogues were also found to be synergistic when combined with rifampicin (see peptides A13, A17, and A21 in Table 3) via a mechanism involving OM disruption.^159^ A similar study with mastoparan-C, a peptide found in the venom of the European hornet, led to the identification of an analogue termed L7A (Table 3), which also displays synergy via OM perturbation.^139^ Another example of a synergist derived from a toxic peptide is myotoxin II, which is isolated from certain snake venoms. Studies with peptide sequences based on the C-terminus of myotoxin II resulted in peptide S1 (Table 3), which showed a good balance of synergy with vancomycin and low hemolytic activity.^179,180^ Attempts at further improving the S1 peptide involved the introduction of Nal residues at the C-terminus to generate S1-Nal, which exhibited enhanced synergistic activity, and S1-Nal-Nal, which also exhibited enhanced synergistic activity but at the expense of increased hemolytic activity (Table 3).^181−184^

#### 1.3.6 Peptide Synergists Discovered via Library Screening

Guardabassi and co-workers recently reported the development and validation of an assay meant to enable high-throughput screens for identifying OM disruption agents.^185^ To this end, they applied a whole-cell screening platform that allows for detection of OM permeabilization in *E. coli* based on the signal generated by a chromogenic substrate reporter for a cytoplasmic β-galactosidase. To validate the assay, a library of peptides and peptidomimetics was screened, which generated a notable hit termed peptide **79** that showed potentiation of various antibiotics at therapeutically relevant levels (Table 3).^185^ In a follow-up study, the same group went on to develop two improved synergists, termed peptides **1** and **2**, along with the all d-amino acid variants, which were also found to effectively potentiate rifampicin against *K. pneumoniae* (Table 3).^71,185^

#### 1.3.7 Peptide Synergists from Phage Display

Phage display techniques have also been applied to identify novel peptides capable of interaction with the OM. In one such investigation, a phage library displaying random 12-mer peptides was screened for the ability to bind to the cell surface of Gram-negative bacteria.^186^ Specificity for the Gram-negative OM was ensured by removal of peptides binding to Gram-positive bacteria by pre-incubation of the library with *Staphylococcus aureus*.^186^ This approach led to the identification of a peptide termed EC5 that exhibits moderate antibacterial activity against *E. coli* and *P. aeruginosa*, with MICs in the range of 8–16 μg/mL against both.^186^ The EC5 peptide was shown to cause OM disruption and cytoplasmic membrane depolarization while exhibiting very little hemolytic activity.^186^ Subsequent synergy studies showed that the peptide was also capable of potentiating the activity of erythromycin, clarithromycin, and telithromycin against *P. aeruginosa*.^131^

#### 1.3.8 Rationally Designed Peptide Synergists

Inspired by the structure of DAPB (see Figure 2), Vaara and co-workers designed a series of linear and cyclic peptides for evaluation as synergists.^187^ The sequences of these peptides were based on an ABB_*n*_ motif, in which A is a basic amino acid and B a hydrophobic residue (see peptides **4** and **5**, Table 3).^187^ Cyclic peptides were also prepared bearing a similar AB_*n*_ motif (see peptide **7**, Table 3).^187^ All peptides were screened for synergistic activity with erythromycin, rifampicin, novobiocin, and fusidic acid, with the rifampicin combinations being the most potent (Table 3).^187^ While the synergistic activity of these peptides could be correlated to their OM-disrupting activity, the effect was not specific, given their high hemolytic activity.^187^

*De novo*-designed peptides have also been explored as a means of generating novel synergists. To this end, the Sahal group developed a number of peptides incorporating key elements found in AMPs and synergists, including amphipathicity, positive charge, and helical conformation.^188,189^ Of note was the introduction of α,β-didehydrophenylalanine (ΔF) into the peptides as a means of constraining the helical conformation of the peptides.^190−192^ Using this approach, two peptides termed ΔFm and ΔFmscr were identified as effective synergists with low toxicity toward mammalian cells (Table 3).

In another recent approach to identifying novel peptide synergists, Yu and colleagues reported the construction of a small library wherein amphipathic peptides where subjected to a proline-scanning strategy to generate novel hinged peptides.^193^ Such proline-hinged peptides are reported to have lower toxicity toward mammalian cells, given that their membrane binding is reduced compared to that of conventional AMPs with a high α-helical conformation.^194^ Proline scanning of two model peptides, LK (LKKLLKLLKKLLKL) and KL (KLLKLLKKLLKLLK), provided a set of peptides that were screened for synergistic activity, with the four most potent peptides displayed in Table 3. The peptides were also screened for hemolysis, which led to identification of peptide KL-L9P as the most promising hit. This peptide was subsequently shown to permeabilize the OM, as evidenced by uptake of *N*-phenylnaphthalen-1-amine (NPN), and was also found to bind LPS without disturbing the inner membrane.^193^ Mouse sepsis studies were also performed to evaluate the *in vivo* synergistic effect of KL-L9P, which displayed a significant potentiation of a number of clinically used antibiotics and resulted in improved overall survival.^193^

In another recently reported study, Zeng et al. described the application of rational design approaches to generate novel helix-forming AMPs based on cytolytic peptide toxins produced by highly virulent strains of *S. aureus*.^195,196^ The peptides thus obtained were shown to have improved physicochemical properties and antibacterial activity, while maintaining low hemolytic activity and cytotoxicity. Among the 16-mers thus generated, two peptides, termed zp12 and zp16, were also found to exhibit potent synergy (Table 3). Notable in this regard is the finding that peptide zp16 specifically potentiates the effect of the glycopeptide antibiotics vancomycin and teicoplanin against highly pathogenic *K. pneumoniae*.^196^ The vancomycin-zp16 combination exhibits negligible toxicity *in vitro* and *in vivo*, and mechanistic studies indicate that zp16 enhances vancomycin’s cell permeability, leading to markedly reduced biofilm formation and rapid bactericidal effect.^196^

In 2022, the group of Ni reported the potentiation of multiple antibiotics, including rifampicin, by two rationally designed peptides named K4 and K5 (Table 3).^197^ These peptides were selected from a library of variants all containing a repeating motif, (WRX)_*n*_, wherein X represents I, K, L, F, and W.^198^ Hemolysis and cytotoxicity assays led to the selection of peptides K4 and K5 as leads.^198^ The finding that these peptides permeabilize the OM resulted in follow-up studies on the potentiation of antibiotics against Gram-negative bacteria.^197^ Apart from synergy, a 15-day resistance assay was also performed for the K4 and K5 peptides, with or without antibiotics, showing no significant resistance development.^197,198^ Also of note, while the inherent activity of K4 was found to be comparable to that of PMB, K4 was reported to display no *in vivo* toxicity when tested as high as 40 mg/kg, while all mice dosed with PMB at the same concentration died within 24 h.^198^

### 1.4 Lipopeptide Synergists

In addition to the exclusively peptide-based synergists described above, lipopeptides have also been explored as synergists. We here cover examples of lipopeptides that do not possess potent inherent antibacterial activity but rather have the capacity to effectively potentiate the activity of other antibiotics. A recent example includes the synthetic paenipeptins developed by Huang and co-workers.^199^ The design of these lipopeptides is based on peptides produced by *Paenibacillus sp.* strain OSY-N that contain a number of unnatural and d-amino acids. Using low hemolytic activity as a selection criterion, a subset of these lipopeptides were selected and screened for synergistic activity. This led to the identification of paenipeptins **1**, **9**, **15**, and **16**, which exhibit potent synergy (Table 3).^199,200^ These lipopeptides were further shown to have OM-disrupting activity, as indicated by the NPN assay. Furthermore, in a murine thigh infection model, paenipeptin 1 was shown to effectively potentiate the *in vivo* activity of both clarithromycin and rifampin against polymyxin-resistant *E. coli*.^200^

**Figure 4:**
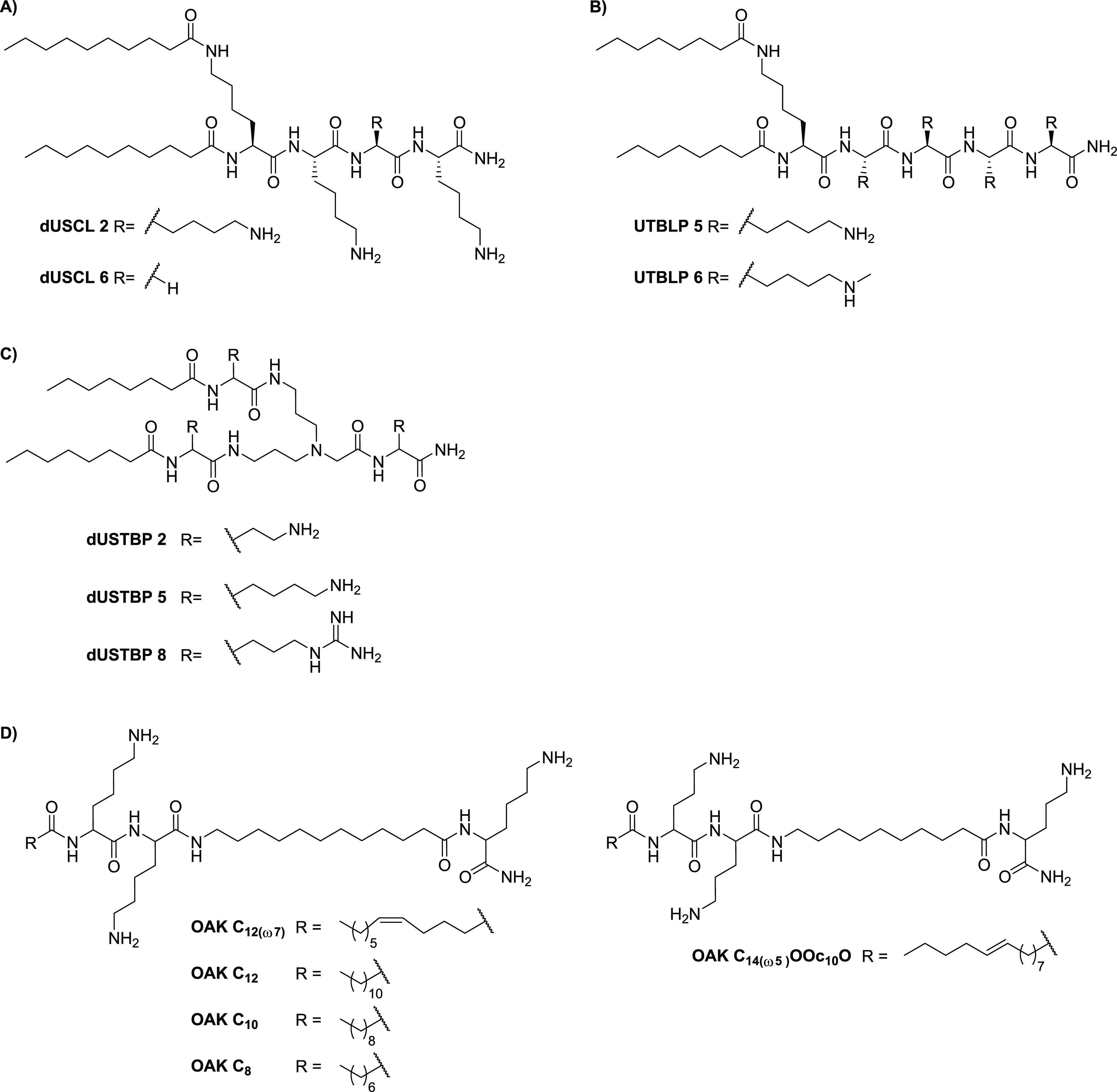
Lipopeptide and lipopeptidomimetic synergists. Representative structures of (A) dilipid ultrashort cationic lipopeptides (dUSCLs), (B) ultrashort tetrabasic lipopeptides (UTBLPs), (C) dilipid ultrashort tetrabasic peptidomimetics (dUSTBPs), and (D) oligo-acyl-lysyls (OAKs).

Small cationic lipopeptides have also been explored as synergists, with the aim of identifying smaller, less hemolytic agents. To this end, Schweizer and co-workers recently reported a series of dilipid ultrashort cationic lipopeptides (dUSCLs) capable of enhancing the activity of clinically used antibiotics against Gram-negative bacteria.^201^ The design of these dUSCLs consists of lysine-rich tetrapeptides bearing various lipids at the N-terminal residue, as illustrated in Figure 4A. It was found that dUSCLs bearing lipids of ≥11 carbon atoms caused significant hemolysis. However, analogues with slightly shorter lipids were found to achieve an acceptable balance of low hemolytic activity and synergistic activity. This led to the identification of dUSCLs **2** and **6** as the most promising synergists (Table 3) capable of sensitizing a range of Gram-negative strains to various antibiotics. The authors also noted that, in addition to permeabilizing the OM, the dUSCLs may also function by indirectly disrupting antibiotic efflux.^201^

The Schweizer group also recently reported a series of ultrashort tetrabasic lipopeptides (UTBLPs) synergists.^202^ These compounds were specifically prepared to assess the effect of lysine *N*-ζ-methylation on the potentiation of antibiotics, inspired by reports suggesting that *N*-methylation can lead to reduced hemolysis, increased proteolytic stability, and improved antibacterial activity.^203−205^ Compared to the dUSCLs, UTBLPs **5** and **6** contain an extra lysine, while an octanoyl group was employed as the lipophilic moiety (Figure 4B).^201,202^ Methylation of the lysine side chain resulted in a reduction of potentiation for rifampicin and novobiocin in both wild-type and resistant Gram-negative strains.^202^ A correlation between the number of methyl groups and loss of activity was seen, while the increase in NPN fluorescence of the trimethylated UTBLPs was on par with that of their un- or monomethylated analogues.^202^

### 1.5 Lipopeptidomimetic Synergists

The Schweizer group also expanded the scope of their dUSCLs by exploring a series of dilipid ultrashort tetrabasic peptidomimetics (dUSTBPs) as proteolytically stable alternatives.^206^ In a focused SAR study, they prepared dUSTBPs consisting of three basic amino acids separated by a molecular scaffold, bis(3-aminopropyl)glycine, along with ligation to simple fatty acids (see Figure 4C).^206^ This led to identification of a number of dUSTBPs capable of potentiating the activity of several antibiotics against pathogenic Gram-negative bacteria while exhibiting low hemolytic activity (Table 3). In particular, dUSTBP **8**, consisting of three l-arginine units and a dilipid eight carbons long, was found to potentiate novobiocin and rifampicin against multi-drug-resistant (MDR) clinical isolates of *P. aeruginosa*, *A. baumannii*, and *Enterobacteriaceae* species.^206^

In 2007, Mor and co-workers introduced the oligo-acyl-lysyls (OAKs) as peptidomimetics of the antimalarial peptide dermseptin S3 (Figure 4D) that were initially evaluated primarily for antimicrobial activity.^207−209^ Among the first series of analogues prepared, OAK C_12(ω7)_ was found to adhere to the OM with minimal insertion, and its antibacterial activity against Gram-negative bacteria improved in combination with ethylenediaminetetraacetate (EDTA).^209−211^ The introduction of a double bond in OAK C_12(ω7)_ resulted in a significant reduction of hemolytic activity compared to that of OAK C_12_, while the slightly less hydrophobic OAK C_10_ and OAK C_8_ analogues also showed no hemolytic activity.^209,212^ In 2013, these four OAKs, as well as the more recently described OAK C_14(ω5)_OOc_10_O, containing ornithine instead of lysine (Figure 4D), were reported to potentiate rifampicin against Gram-negative bacteria (Table 3).^212,213^ Interestingly, the synergistic activity of the OAKs was maintained in human plasma but was suppressed by addition of anti-complement antibodies, suggesting that these compounds sensitize Gram-negative bacteria to the action of antibacterial innate immune mechanisms.^213^

## 2 Cationic Steroids

**Figure 5:**
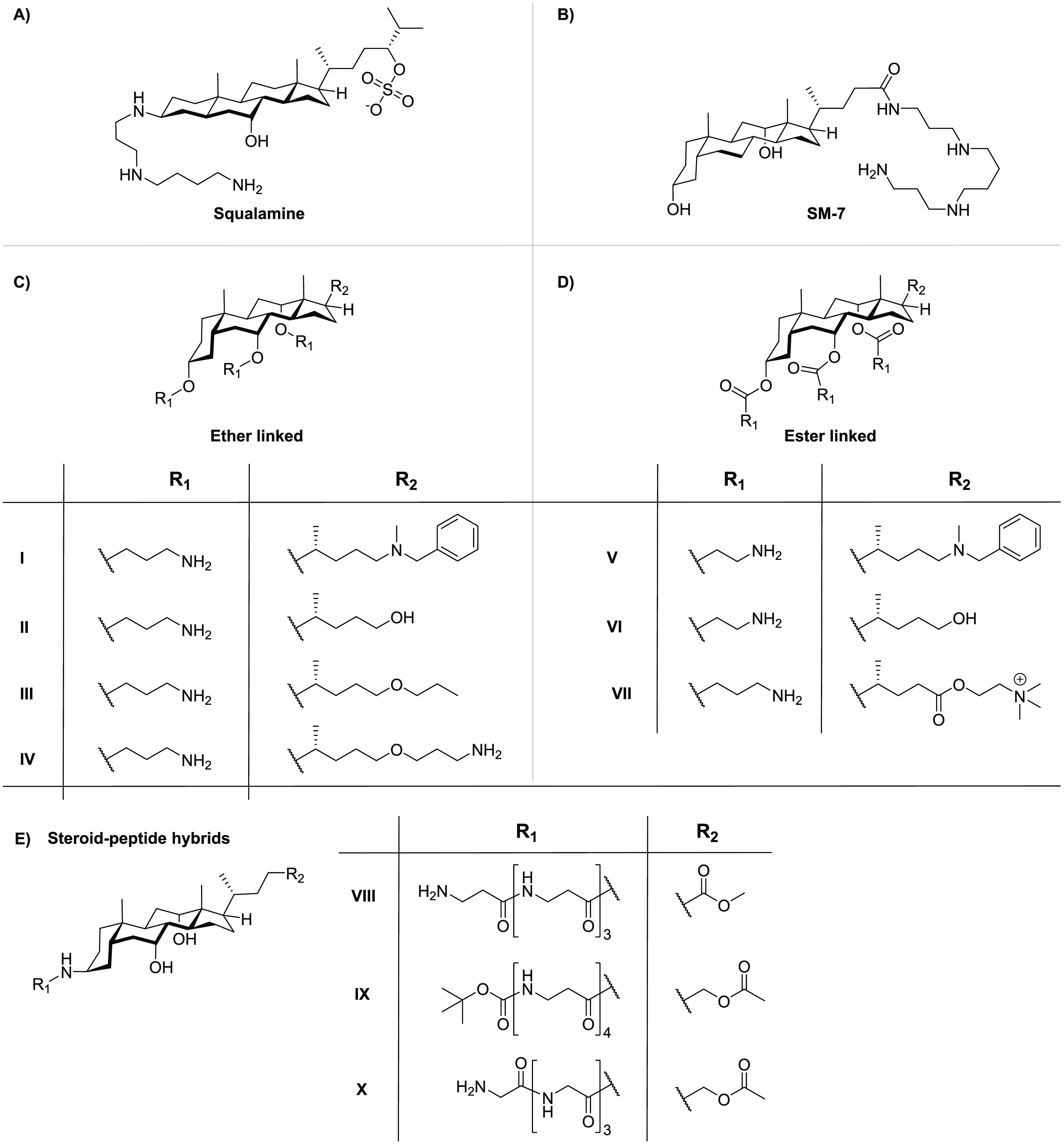
Overview of the synergistic steroids (A) squalamine, (B) squalamine mimic SM-7, (C) polycationic cholic acid ether-linked steroid synergists, (D) polycationic cholic acid ester-linked steroid synergists, and (E) steroid–peptide hybrids.

In 1993, the isolation of squalamine from tissues of the dogfish shark *Squalus acanthias* was reported.^224^ Squalamine consists of a steroid core linked to a spermidine moiety (Figure 5A) and was found to exhibit broad antimicrobial activity.^224^ Later, it was established that squalamine disrupts membranes and is also hemolytic. Notably, investigations into its synergistic activity showed that it was unable to potentiate erythromycin against wild-type strains, showing an effect only against a *P. aeruginosa* strain overproducing MexAB-OprM efflux pumps (see Table 4).^225,226^ A few years after its discovery, novel squalamine mimics (SMs) were synthesized in an attempt to enhance antibacterial activities (Figure 5B).^227^ These synthetic analogues consist of cholic and deoxycholic acid as the steroid backbone to which a spermidine chain is appended. This approach resulted in the identification of analogue SM-7, which was found to potentiate rifampicin against multiple Gram-negative bacteria (Table 4).^227^ However, like squalamine, SM-7 also possesses significant hemolytic activity, limiting its potential for systemic use.^227^

**Table 4:** Overview of Synergists Based on Cationic Steroids

name	ref	FICI	pathogen	antibiotic	hemolytic activity^a^
squalamine	(224, 226)	0.35^b^	*P. aeruginosa*	erythromycin	>10% (10 min)
SM-7	(227)	0.063	*K. pneumoniae*	rifampicin	<10% (24 h)

**Polycationic Cholic Acid Analogues**					
*Ether-linked*					
**I**	(229, 230)	0.035	*K. pneumoniae*	rifampicin	>10% (24 h)
**II**	(230)	0.029	*K. pneumoniae*	novobiocin	<10% (24 h)
**III**	(230)	0.022	*K. pneumoniae*	novobiocin	>10% (24 h)
**IV**	(232)	0.13	*K. pneumoniae*	rifampicin	<10% (24 h)
*Ester-Linked*					
**V**	(233)	0.057^b^	*E. coli*	erythromycin	NR
**VI**	(233)	0.064^b^	*E. coli*	erythromycin	NR
**VII**	(234)	0.176^b^	*E. coli*	erythromycin	<10% (24 h)
*Steroid–Peptide Hybrids*					
**VIII**	(239)	0.099	*E. coli*	erythromycin	NR
**IX**	(239)	0.093	*E. coli*	erythromycin	NR
**X**	(239)	0.078	*E. coli*	erythromycin	NR

a Non-hemolytic is defined as <10% hemolysis compared to positive control, with incubation times denoted in parentheses; NR denotes no data reported.

b FICI calculated from MIC values reported in the cited literature references.

In another approach, the Savage group also employed the cholic acid backbone but with the aim of mimicking polymyxins through the amphiphilic positioning of positive charges (Figure 5C,D).^228,229^ In doing so, a variety of cationic steroids were developed and screened for inherent antimicrobial activity as well as the capacity to potentiate antibiotics against Gram-negative bacteria.^229−237^ The hydroxyl groups on the cholic acid backbone provide convenient functionalities for the incorporation of positively charged moieties via formation of ether (Figure 5C) or ester (Figure 5D) linkages. Among the ether-linked series, an analogue bearing three carbon atom spacers between the steroid and the primary amine groups, along with an *N*-benzylated tertiary amino group at the C24 position (analogue **I**, Figure 5C), was found to exhibit both inherent antimicrobial activity and synergistic activity.^229^ Interestingly, replacement of the lipophilic *N*-benzyl moiety with a hydroxyl group led to analogue **II**, which showed a significant reduction of inherent activity while maintaining a strong ability to potentiate the activity of erythromycin against *E. coli*.^228,229^ The decreased lipophilicity of analogue **II** also reduced the hemolytic activity seen with analogue **I** (Table 4). Follow-up studies revealed that conversion of the free hydroxyl group at the C24 position to the propyl ether, as in analogue **III**, significantly increased the hemolytic activity.^230,231^ Notably, addition of a terminal amino group to the propyl ether moiety provided analogue **IV**, which exhibited significantly reduced hemolysis relative to that of analogue **III** while maintaining effective synergistic activity (Table 4).^232^ A series of ester-linked analogues were also prepared by the Savage group (Figure 5D), wherein compounds **V**, **VI**, and **VII** exhibited synergistic activity comparable to that of the corresponding ether variants (Table 4).^233,234^ Amide analogues were also explored; however, they exhibited a significant lower potentiation of erythromycin, presumably due to conformational constraints, relative to the more active esters.^233^

In addition to the polycationic steroids described above, steroid–peptide hybrids have also been explored as synergists.^237−239^ In one case, Bavikar et al. reported a series of hybrids wherein simple tetrapeptides were coupled to cholic acid in an attempt to mimic the squalamine tail (Figure 5E).^239^ As indicated in Table 4, these steroid–peptide hybrids exhibit potent synergy with erythromycin against *E. coli*. While the hemolytic activity of these compounds was not reported, they were described as having low cytotoxicity toward HEK293 and MCF-7 cells.^239^

## 3 Non-steroid Small-Molecule Synergists

### 3.1 Synergists Based on Approved Drugs

**Figure 6:**
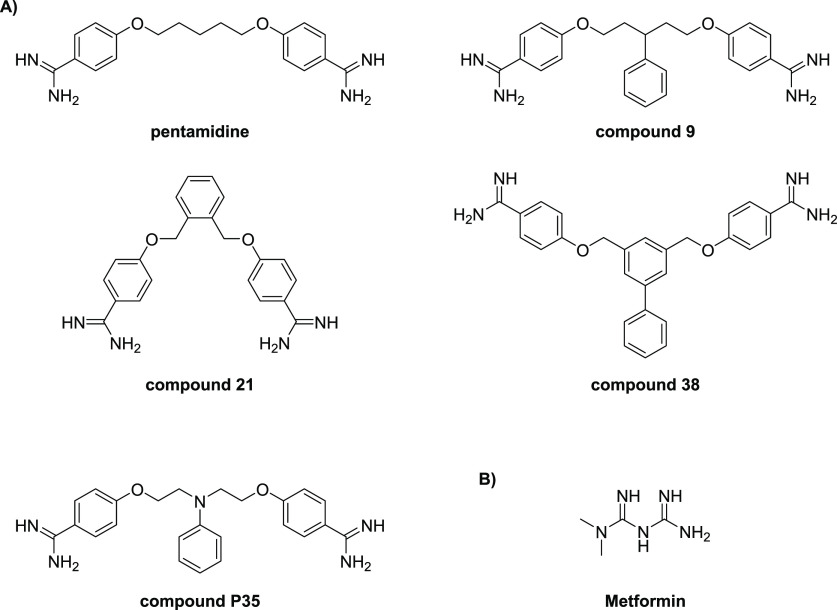
Representative structures of recently reported (A) bis-amidine synergists and (B) metformin.

Recently, Brown and co-workers reported an innovative screening platform for the identification of non-lethal, OM-active compounds with potential as adjuvants for conventional antibiotics.^240^ They applied their screen to a library of 1440 previously approved drugs, which resulted in the identification of three hits. Among the three hits identified, the anti-protozoal agent pentamidine (Figure 6A) was subsequently found to display the highest synergistic potency (Table 5).^240^ Notably, while pentamidine’s OM-targeting mechanism was found to be driven by interaction with LPS, *mcr-*resistance did not affect its synergistic potential.^240^ The potentiation of novobiocin by pentamidine was also established *in vivo* against wild-type and resistant *A. baumannii*.^240^ Subsequently, a focused SAR study using commercially available bis-amidines similar in structure to pentamidine led to the identification of compound **9** as an even more potent synergist (Figure 6a and Table 5).^240^

**Table 5:** Overview of Non-steroid Small-Molecule Synergists

name^a^	ref	FICI	pathogen	antibiotic	hemolytic activity^b^
**Synergists Based on Approved Drugs**					
pentamidine	(240, 241)	0.25	*E. coli*	rifampicin	<10% (20 h)
compound 9	(240, 241)	<0.047	*E. coli*	rifampicin	>10% (20 h)
compound 21	(241)	≤0.094	*E. coli*	rifampicin	<10% (20 h)
compound 38	(241)	≤0.039	*E. coli*	rifampicin	>10% (20 h)
compound P35	(242)	0.094	*A. baumannii*	novobiocin	<10% (45 min)^c^
metformin	(245)	0.375	*E. coli*	vancomycin	<10% (1 h)

**High-Throughput Screening Hits**					
MAC-0568743	(246)	≤0.16	*E. coli*	rifampicin	NR
liproxstatin-1	(246)	0.25^d^	*E. coli*	rifampicin	NR
BWC-Aza1	(247)	0.258	*E. coli*	rifampicin	<10% (45 min)
BWC-Aza2	(247)	0.06	*A. baumannii*	rifampicin	<10% (45 min)

**Peptidomimetics**					
OAK C_12(ω7)_	(212)	≤0.073^d^	*E. coli*	rifampicin	>10% (3 h)
OAK C_12_	(212)	≤0.211^d^	*E. coli*	rifampicin	>10% (3 h)
OAK C_10_	(212)	≤0.036^d^	*E. coli*	rifampicin	<10% (3 h)^c^
OAK C_8_	(212)	≤0.078^d^	*E. coli*	rifampicin	<10% (3 h)^c^
C_14(ω5)_OOC_10_O	(213)	0.20^d^	*K. pneumoniae*	rifampicin	<10% (3 h)^c^
dUSTBP 2	(206)	≥0.250	*P. aeruginosa*	rifampicin	<10% (1 h)
dUSTBP 5	(206)	≥0.125	*P. aeruginosa*	rifampicin	<10% (1 h)
dUSTBP 8	(206)	≥0.002	*A. baumannii*	novobiocin	<10% (1 h)

**Synergists with a Polyamine Motif**					
d-LANA-14	(249, 250)	0.09	*P. aeruginosa*	rifampicin	<10% (1 h)
naphthylacetylspermine	(251)	0.125^d^	*E. coli*	novobiocin	nr
bisacyl-homospermine 8a	(253)	0.304^d^	*E. coli*	rifampicin	<10% (30 min)
bisacyl-homospermine 8b	(253)	0.297^d^	*E. coli*	rifampicin	>10% (30 min)
spermidine analogue 14	(258)	0.255^d^	*E. coli*	erythromycin	<10% (1 h)^c^
spermidine analogue 17	(258)	0.255^d^	*P. aeruginosa*	erythromycin	<10% (1 h)^c^
600-Da BPEI	(261, 275)	0.26	*P. aeruginosa*	erythromycin	<10% (1 h)

**Plant-Derived Synergists**					
eugenol	(262, 276)	≤0.2^d^	*P. aeruginosa*	rifampicin	<10% (24 h)
linalool	(263, 277)	0.37	*E. coli*	erythromycin	<10% (4 h)
thymol	(271, 278)	0.25	*E. coli*	erythromycin	<10% (1 h)
cinnamaldehyde	(271, 279)	0.24	*E. coli*	erythromycin	<10% (48 h)
*trans*-cinnamic acid	(272, 280)	0.36	*E. coli*	erythromycin	<50% (1 h)
ferulic acid	(272, 280)	0.48	*E. coli*	erythromycin	<50% (1 h)
3,4-dimethoxycinnamic acid	(272, 280)	0.42	*E. coli*	erythromycin	<50% (1 h)
2,4,5-trimethoxycinnamic acid	(272, 280)	0.22	*E. coli*	erythromycin	<50% (1 h)

a Compound names are provided as given in the cited literature references.

b Non-hemolytic is defined as <10% hemolysis compared to positive control, with incubation times denoted in parentheses; NR denotes no data reported.

c Concentration tested was lower than 100 μg/mL.

d FICI calculated from MIC values reported in the cited literature references.

Inspired by these findings, our group recently undertook a broad SAR investigation wherein a number of structurally unique bis-amidines were synthesized and evaluated as synergists.^241^ Specifically, we focused our attention on the length and rigidity of the linker motif as well as the geometry of the amidine groups on the aromatic rings. In addition to assessing the synergistic activity of the new bis-amidines prepared, we also performed hemolysis assays with each compound to ascertain OM selectivity. Given the potent synergy previously reported for bis-amidine **9**,^240^ we also synthesized it to use as a benchmark. Among the compounds prepared in our study, bis-amidine **21**, containing an *ortho*-substituted benzene linker, was found to be significantly more synergistic than pentamidine and displayed no hemolytic activity (Figure 6A and Table 5).^241^ We also found that the introduction of additional aromatic groups to the linker, such as in compound **38**, led to further enhancement of synergy; however, this came at the cost of increased hemolytic activity (Table 5). Interestingly, our studies also revealed benchmark bis-amidine **9** to be hemolytic. These findings further highlight the importance of assessing OM selectivity when pursuing synergists.^241^

The Brown group also recently reported a follow-up SAR study aimed at further enhancing the therapeutic potential of bis-amidine synergists.^242^ Similar to our own SAR study, the rigidity, conformational flexibility, and lipophilicity were further explored. In addition, the roles of chirality and charge were also investigated.^242^ A key focus of this study was to identify bis-amidine synergists with improved off-target effects relative to those of pentamidine, especially the QT prolongation resulting from its effect on the hERG ion channel.^242−244^ This led to compound **P35**, which was shown to have the same synergistic mode of action as pentamidine; it displayed a strong potentiation of novobiocin and no hemolytic activity (Table 5). Furthermore, compound **P35** outperformed pentamidine on multiple levels: an improvement in cytoxicity, a higher efficacy in a mouse infection model, and reduced hERG inhibition.^242^

Wang and co-workers also recently reported a study wherein the Prestwick Chemical Library, comprising 158 FDA-approved drugs, was assessed for compounds exhibiting synergy with doxycycline.^245^ This led to the finding that metformin, a commonly prescribed anti-diabetic agent (Figure 6B), effectively potentiates vancomycin as well as tetracycline antibiotics, particularly doxycycline and minocycline, against MDR *S. aureus*, *Enterococcus faecalis*, *E. coli*, and *Salmonella enteritidis*.^245^ The capacity for metformin to disturb the OM was assessed using the NPN assay, revealing an increase in *E. coli* OM permeability in a dose-dependent manner. Of particular note was the finding that metformin was also able to fully restore the activity of doxycycline in animal infection models.^245^

### 3.2 Small-Molecule Synergists via High-Throughput Screening

**Figure 7:**
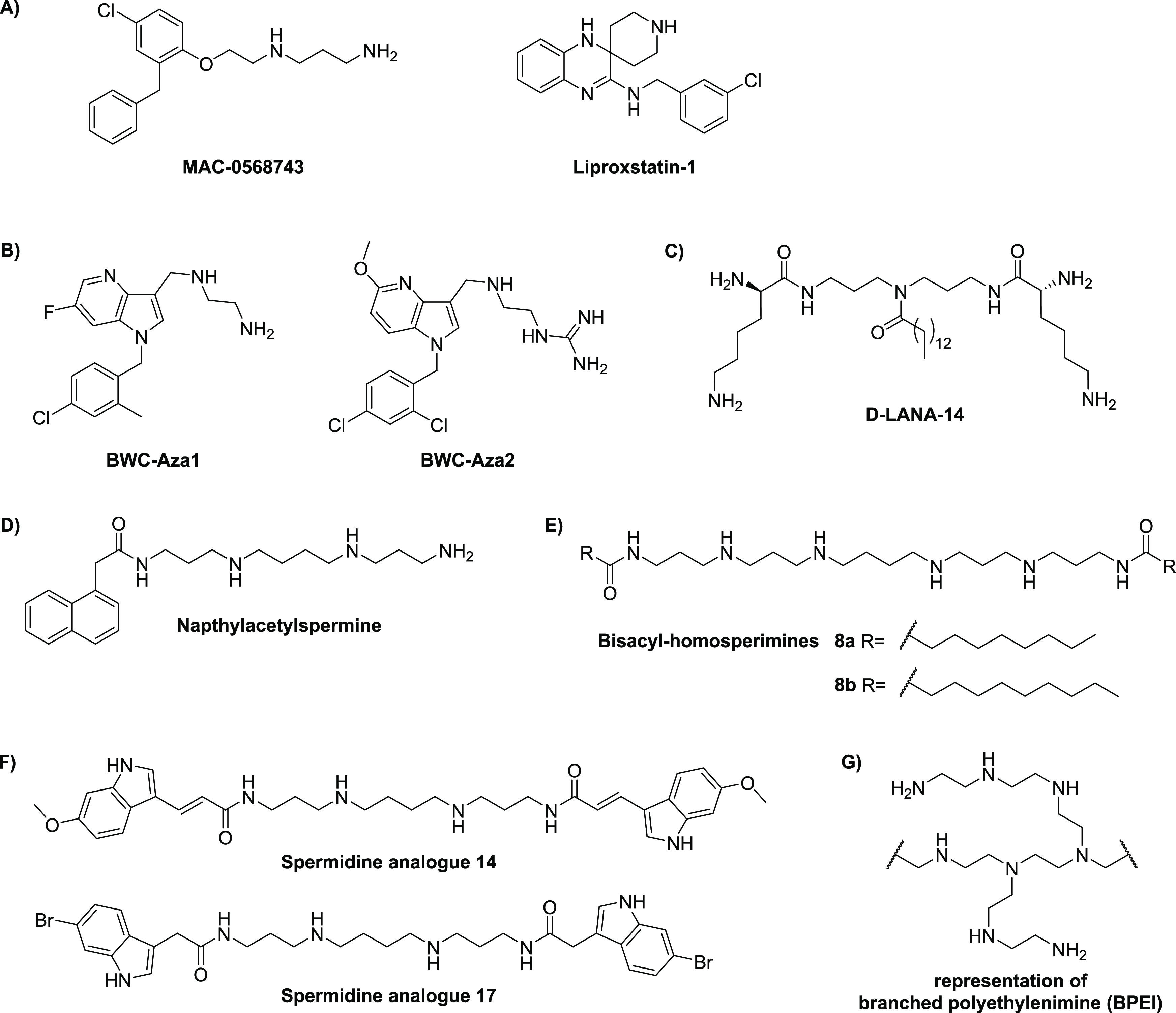
Non-steroid small-molecule synergists: (A) synergists identified via HTS, (B) azaindole synergists, (C) d-LANA-14 based on a norspermidine core linked to two d-lysine residues and a central tetradecanoyl moiety, (D) joro spider toxin-inspired naphthylacetylspermine, (E) bisacyl-homospermines, (F) indole-3-acrylamidospermine conjugates, and (G) representation of 600 Da branched polyethylenimine (BPEI).

Following the success in applying their OM perturbation reporter assay to identify pentamidine as a potent synergist, the Brown group applied the same approach in a much larger high-throughput screening (HTS) campaign with a library of ca. 140 000 synthetic compounds.^240,246^ This, in turn, led to the identification of 39 hits that were subsequently screened for synergistic activity with rifampicin.^246^ Among these hits, MAC-0568743 and liproxstatin-1 (Figure 7A) were found to be particularly active synergists (Table 5).^246^ Both compounds were found to potentiate the activity of the Gram-positive-targeting antibiotics rifampicin, novobiocin, erythromycin, and linezolid. This potentiation was further shown to be due to selective disruption of the OM, driven by interactions with LPS, and neither compound impacted the inner membrane.^246^

In another recently reported campaign, Datta and co-workers screened a focused library of 3000 drug-like compounds for antibiotic synergy using a whole-cell-based phenotypic assay.^247^ This led to the identification of a series of azaindoles that potentiate the MICs of novobiocin and rifampicin by 100–1000-fold vs Gram-negative bacteria. Optimization studies led to compounds BWC-Aza1 and BWC-Aza2 (see Figure 7B), both of which were screened for synergistic activity with an extensive panel of antibiotics against *E. coli* (Table 5). The OM-permeabilizing activity of the azaindoles was also probed using the NPN assay, revealing dose-dependent disruption.^247^

### 3.3 Small-Molecule Polyamine Synergists

In recent years, the polyamines norspermine and norspermidine have been explored as starting points for the development of antibacterial and antibiofilm agents.^248,249^ Building on this work, the Haldar group recently reported the development of d-LANA-14, composed of a norspermidine core linked to two d-lysines, along with conjugation to a tetradecanoyl chain at the central secondary amine (Figure 7C).^250^d-LANA-14 showed potent synergy with tetracycline or rifampicin against meropenem-resistant *A. baumannii* and *P. aeruginosa* clinical isolates (Table 5) and, importantly, was also found to disrupt established biofilms formed by these pathogens.^250^d-LANA-14 was shown to perturb the OM by means of the NPN assay and, importantly, was also found to exhibit potent *in vivo* activity when combined with rifampicin, resulting in a significant reduction of bacterial burden in a mouse model of burn-wound infection.^250^

In another study involving small-molecule polyamines, Katsu and co-workers investigated synthetic analogues of the joro spider toxin as OM-disrupting agents, leading to the identification of naphthylacetylspermine (Figure 7D), which was found to potentiate the activity of novobiocin against *E. coli* (Table 5).^251^ Mechanistic studies revealed that administration of naphthylacetylspermine causes OM disruption, which was attributed to displacement of LPS-associated Ca^2+^. In addition, naphthylacetylspermine was found to promote cellular uptake of the tetraphenylphosphonium (TPP^+^), indicating membrane permeabilization, a finding similar to that obtained with PMBN.^251,252^ Interestingly, spermidine and spermine were also found to induce loss of Ca^2+^ but did not cause uptake of TPP^+^, pointing to the importance of the naphthyl moiety for membrane permeabilization.^252^ Given that no hemolysis data was reported for naphthylacetylspermine, it is not possible to assess the selectively of its OM activity.

The David group also reported the development of acylated polyamines as LPS neutralizing agents capable of functioning as OM-disrupting synergists.^253−255^ A series of monoacyl- and bisacyl-homospermines were prepared and evaluated as potentiators of rifampicin, resulting in the identification of two potent synergists, compounds **8a** and **8b** (see Figure 7E and Table 5).^253^ A clear correlation between the length of the lipophilic tail and hemolytic activity was seen, with compound **8a** appearing to strike an optimal balance.^253^ Using a similar approach, Copp and co-workers introduced the indole-3-acrylamido-spermine conjugates inspired by a class of indole-spermidine alkaloid natural products.^256,257^ An SAR study led to the development of spermidine analogues like **14** and **17**, which exhibited effective synergy with various antibiotics (Figure 7F and Table 5).^256,258^ These compounds affect bacterial membrane integrity and show low cytotoxicity and hemolytic activity. Interestingly, compound **14** was also found to inhibit bacterial efflux pumps, suggesting that the potentiation of antibiotics by these compounds may be attributed to a dual mechanism of action.^256,258^

Given the inclusion criteria noted in the introduction, only small-molecule synergists (MW under 1500 kDa) are included in this Review, and as such we do not discuss larger polycationic polymers even though some have been shown to exhibit synergistic activity.^90−96,259,260^ It is noteworthy, however, that branched polyethylenimine (BPEI) with a MW of 600 Da shows synergistic activity (Figure 7G, Table 5) and can also eradicate biofilms when co-administered with a variety of antibiotics.^261^ Mechanistic studies using isothermal titration calorimetry and fluorescence spectroscopy indicate that, at the concentration required for antibiotic potentiation, 600 Da BPEI reduces diffusion barriers from LPS without disrupting the OM itself.^261^

### 3.4 Plant-Derived Synergists

**Figure 8:**
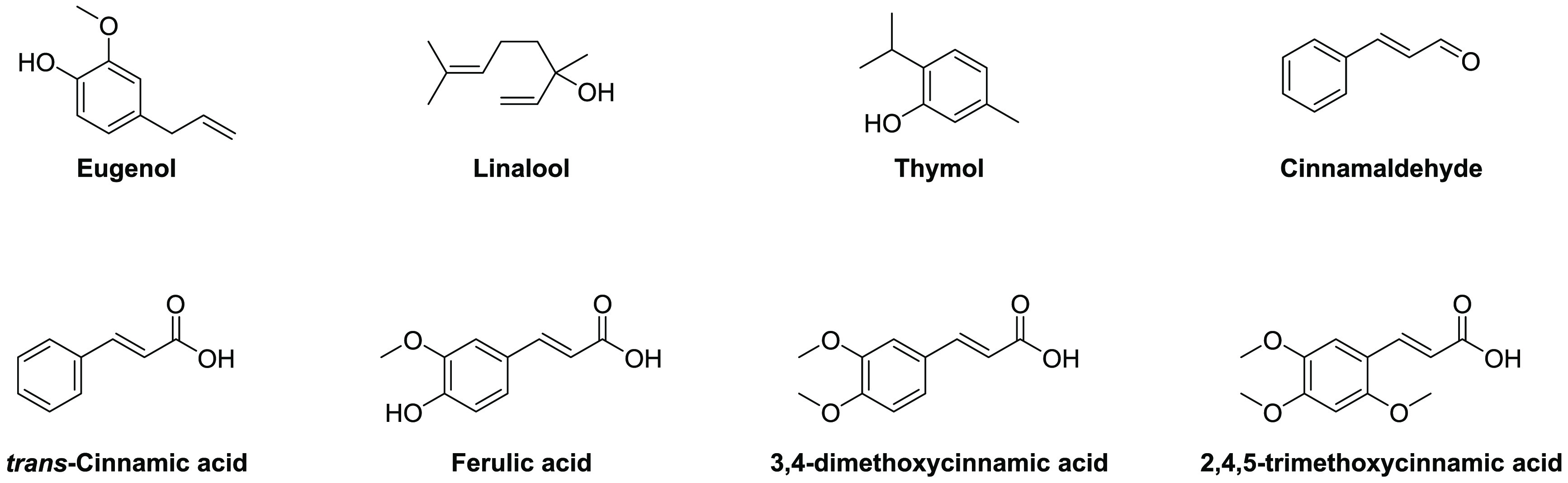
Plant-derived natural products reported to potentiate the activity of antibiotics against Gram-negative bacteria.

A number of plant-derived compounds have also been reported to potentiate the activity of antibiotics against Gram-negative bacteria (Table 5). These include natural products like eugenol, a major component of clove oil; linalool, which can be isolated from coriander; thymol, which is extracted from thyme; and cinnamaldehyde and cinnamic acid, which are found in the bark and leaves of the cinnamon tree (Figure 8).^262−268^ Important to note is that only pure compounds derived from plants are included in our assessment. We refer the reader to other reviews on the synergistic activity of essential oils or crude extracts.^269,270^ Notably, most plant-derived compounds reported to potentiate antibiotics against Gram-negative bacteria are not cationic, setting them apart from most other synergists. Despite their lack of positive charge, a number of investigations have shown that the synergy associated with these compounds is a function of their ability to induce OM permeabilization (Table 6).^262,263,271−273^ The broad range of biological activities associated with cinnamic acid and its derivatives, including ferulic acid, 3,4-dimethoxycinnamic acid, and 2,4,5-trimethoxycinnamic acid (Figure 8), has been recently reviewed including synergistic effects associated with OM disruption.^274^ Interestingly, despite its clear structural similarities with cinnamic acid, studies with cinnamaldehyde suggest that it may operate via a different synergistic mechanism. Unlike cinnamic acid, cinnamaldehyde does not increase OM permeabilization based on the NPN assay, but it does exhibit synergistic effects with erythromycin and novobiocin (Table 5).^271,273^

**Table 6:** Overview of Synergists Based on Clinically Used Antibiotics

name^a^	ref	FICI	pathogen	antibiotic	hemolytic activity^b^
**Tobramycin Derivatives**					
TOB-MOX 1	(291)	0.125	*P. aeruginosa*	novobiocin	<10% (30 min)
tobramycin-ciprofloxacin 1e	(292)	<0.04	*P. aeruginosa*	rifampicin	<10% (30 min)
tobramycin-rifampicin 1	(293)	0.28	*P. aeruginosa*	rifampicin	<10% (1 h)
tobramycin-rifampicin 2	(293)	0.15	*P. aeruginosa*	erythromycin	<10% (1 h)
tobramycin-rifampicin 3	(293)	0.06	*P. aeruginosa*	erythromycin	<10% (1 h)
tobramycin-lysine 3	(294)	0.008	*P. aeruginosa*	novobiocin	<10% (1 h)
TOB-NMP 1	(296)	≥0.008	*P. aeruginosa*	rifampicin	<10% (30 min)
TOB-PAR 2	(296)	≥0.008	*P. aeruginosa*	rifampicin	<10% (30 min)
tobramycin homodimer 1	(297)	0.07	*P. aeruginosa*	novobiocin	<10% (1 h)
tobramycin homodimer 2	(297)	0.08	*P. aeruginosa*	novobiocin	<10% (1 h)
tobramycin homodimer 3	(297)	0.05	*P. aeruginosa*	novobiocin	<10% (1 h)
tobramycin-cyclam 1	(298)	0.13	*P. aeruginosa*	novobiocin	<10% (30 min)
tobramycin-cyclam 2	(298)	0.13	*P. aeruginosa*	novobiocin	<10% (30 min)
tobramycin-cyclam 3	(298)	0.08	*P. aeruginosa*	novobiocin	<10% (30 min)

**Nebramine Derivatives**					
NEB-MOX 1a	(299)	≥0.002	*K. pneumoniae*	rifampicin	NR
NEB-CIP 1b	(299)	≥0.008	*P. aeruginosa*	rifampicin	<10% (1 h)
NEB-NMP 2	(299)	≥0.004	*P. aeruginosa*	rifampicin	NR
nebramine-cyclam	(300)	0.25	*P. aeruginosa*	rifampicin	<10% (1 h)

**Levofloxacin–Polybasic Peptide Conjugates**					
levofloxacin conjugate 10	(301)	0.10	*P. aeruginosa*	rifampicin	<10% (1 h)
levofloxacin conjugate 11	(301)	0.10	*P. aeruginosa*	novobiocin	<10% (1 h)
levofloxacin conjugate 12	(301)	0.08	*P. aeruginosa*	novobiocin	<10% (1 h)

a Compound names are provided as given in the cited literature references.

b Non-hemolytic is defined as <10% hemolysis compared to positive control, with incubation times denoted in parentheses; NR denotes no data reported.

## 4 Antibiotic-Derived Synergists

In general, the antibiotic potentiators discussed above show little to no inherent antibacterial activity. There are, however, a number of reports describing antibacterial compounds that also exhibit OM-disrupting effects and, in doing so, synergize with antibiotics that are otherwise inactive toward Gram-negative bacteria. The synergists described in this section are specifically included on the basis of their OM-disrupting activity rather than a contribution of their inherent activity to synergy. We therefore do not include the combination of rifampicin with imipenem or trimethoprim, which is solely based on functional synergy.^281,282^ In addition, we also do not cover reports describing systems where an OM-perturbing motif like PMBN is covalently linked to another antibiotic as a means of enhancing anti-Gram-negative activity.^39,283−285^

### 4.1 Tobramycin-Derived Synergists

**Figure 9:**
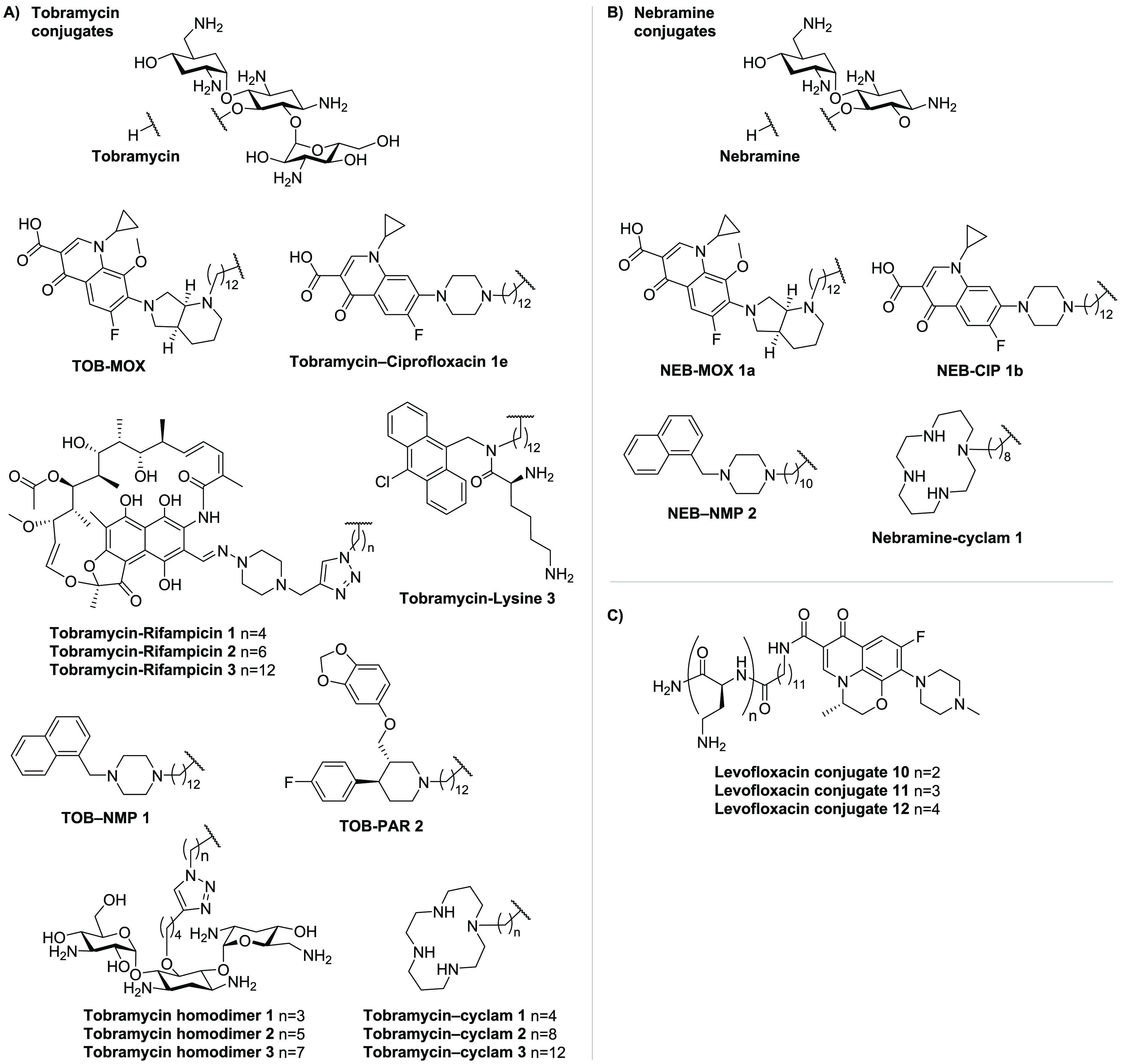
Synergists based on clinically used antibiotics: (A) tobramycin (TOB) conjugates, (B) nebramine (NEB) analogues, and (C) polybasic conjugated levofloxacin hybrids.

Tobramycin (Figure 9A) belongs to the aminoglycoside class of antibiotics that function by inhibiting ribosomal protein synthesis in bacteria. Recent studies have also revealed that aminoglycosides like tobramycin also interact with bacterial membranes by specifically binding to LPS and, in doing so, cause membrane depolarization.^286−290^ Building on these insights, Schweizer and co-workers prepared and assessed a number of conjugates wherein one tobramycin molecule is linked to a second antibiotic, providing hybrid systems that possess both inherent antibacterial activity and potent synergy with other antibiotics (Figure 9A).^291−294,283,295−301^ Among the first hybrids prepared was a series tobramycin–fluoroquinolone conjugates.^291,292^ Both the optimal sites of conjugation and linker lengths between the two antibiotics were investigated, revealing TOB-MOX, a tobramycin–moxifloxacin hybrid, and tobramycin–ciprofloxacin conjugate **1e** to be potent synergists (Table 6).^292^ OM disruption was confirmed for both hybrids using the NPN assay, and both were found to potentiate multiple antibiotics, including rifampicin, erythromycin, novobiocin, and vancomycin.^291,292^ Also of note was the finding that these hybrids exhibited a significantly reduced capacity to inhibit protein translation compared to that of tobramycin.^291,292^ Conversely, the hybrids were found to maintain, and in some cases exceed, the gyrase-inhibiting activity of the parent fluoroquinolones.^291,292^ Another series of hybrids was prepared by coupling tobramycin with rifampicin, which targets the bacterial RNA polymerase.^293^ As for the fluoroquinolone conjugates, the inherent activity of the tobramycin–rifampicin conjugates was significantly reduced compared to that of the parent antibiotics. Again, however, some hybrids were found to exhibit synergy via an OM-disrupting mechanism (see tobramycin–rifampicins **1**–**3**, Figure 9A).^292−294,302^

A number of other hybrids have also been reported by the Schweizer group wherein tobramycin was coupled to various other small molecules known to engage with different bacterial targets. In one case, tobramycin was coupled to a lysine-based amphiphile known to function as a membrane permeabilizer (see tobramycin–lysine **3**, Figure 9A).^294,303^ This conjugate was found to effectively potentiate the activity of novobiocin, erythromycin, and vancomycin (Table 6).^294,304^ The same group also explored hybrids wherein tobramycin was coupled to small-molecule efflux pump inhibitors such as 1-(1-naphthylmethyl)piperazine (NMP) and paroxetine (PAR) (Figure 9A).^45,295,305−307^ Along with potent synergy against *P. aeruginosa* (Table 6), these hybrids were also found to cause OM disruption and inner membrane depolarization.^295,296^ Two additional generations of tobramycin conjugates were also reported: tobramycin homodimers and tobramycin coupled to chelating cyclams (Figure 9A).^297,298^ The dimerization of tobramycin was conveniently achieved by means of copper-catalyzed azide–alkyne click chemistry, resulting in potent synergists that also exhibit enhanced OM disruption relative to tobramycin itself (Table 6).^297^ A combination of novobiocin and tobramycin homodimer **1** (both administered at 50 μg/mL) was further shown to have *in vivo* efficacy against *A. baumannii* in a wax worm larvae model.^297^ Studies with the corresponding monomeric tobramycin azide and alkyne precursors revealed neither to be synergistic, underscoring the need for dimerization to achieve synergy.^297^ In the case of the tobramycin–cyclam conjugates, the introduction of the cyclam chelating group was hypothesized to aid in the OM permeabilization by sequestration of divalent cations bridging the Lipid A phosphate groups.^298,308−310^ While tobramycin–cyclam hybrids **1**–**3** effectively potentiated novobiocin, rifampicin, vancomycin, and erythromycin (Table 6), it is also particularly noteworthy that they also enhanced the activity of meropenem against both carbapenem-resistant and -sensitive strains.^298^ This effect was abrogated by the addition of excess MgCl_2_, further supporting a mode of action driven by OM disruption.^298^

### 4.2 Nebramine-Derived Synergists

Following on their work with tobramycin hybrids, the Schweizer group also prepared a number of analogous nebramine conjugates (Figure 9B). Nebramine (NEB) is a disaccharide sub-unit of tobramycin that interestingly displays activity against tobramycin-resistant strains and also interacts with the OM.^287,311−317^ The NEB hybrids synthesized included conjugates with moxifloxacin (MOX), ciprofloxacin (CIP), NMP, and cyclam (Figure 9B).^299,300^ These hybrids were all found to effectively potentiate the activity of multiple classes of antibiotics against a range of Gram-negative bacteria (Table 6). Furthermore, NEB-MOX 1a, NEB-CIP 1b, and NEB-NMP 2 were also reported to dissipate proton motive force and proposed to cause OM disruption, as for the corresponding tobramycin conjugates.^291,294,295,299,300^

### 4.3 Levofloxacin–Polybasic Peptide Conjugates as Synergists

Schweizer and co-workers also recently reported another class of antibiotic-based synergists, consisting of levofloxacin conjugated to polybasic peptides of varying lengths (Figure 9C).^301^ While these levofloxacin–peptide hybrids were found to be non-hemolytic, they were also shown to be essentially devoid of inherent antimicrobial activity (MICs typically >128 μg/mL). They did, however, exhibit strong potentiation of numerous antibiotics against MDR clinical isolates of *P. aeruginosa*, *E. coli*, *K. pneumoniae*, and, to a lesser extent, *A. baumannii* (Table 6).^301^ Preliminary mechanistic studies indicate that these conjugates potentiate other antibiotics both by blocking active efflux and by permeabilization of the OM.^301^

## 5 Chelating Agents as OM-Disrupting Synergists

**Figure 10:**
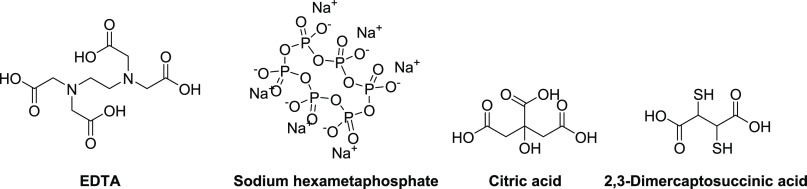
Chelating agents with demonstrated synergistic activity.

The activity of antibiotics can also be potentiated by chelating agents that disturb the integrity of the OM by sequestering the divalent cations Mg^2+^ or Ca^2+^ coordinated by the phosphate groups of the lipid A core of LPS (Figure 1B).^32^ The pre-eminent chelating agent, EDTA (Figure 10), is a well-described synergist, and its reported ability to potentiate antibiotics actually pre-dates the reported synergistic activity of PMBN.^49,318−321^ Exposure of Gram-negative bacteria to EDTA is accompanied by the significant release of LPS and, as for treatment with PMBN, also results in the increased uptake of NPN.^322−324^ While the potentiating effects of EDTA on antibiotics such as novobiocin and rifampicin are well documented, FICI values have not been reported in literature and cannot be readily calculated from published data.^320,321,323,325^ Similarly, for the other chelating agents here discussed, no FICI values could be found in the literature, and, as such, we do not provide a summary table as was done for the other synergists discussed in this Review.

In additional to his seminal work with PMBN, Vaara also reported the potentiation of hydrophobic antibiotics by sodium hexametaphosphate (HMP, Figure 10) against Gram-negative bacteria as well as the increase in NPN uptake in cells treated with this potent Ca^2+^-binding agent.^326^ In a similar study, Ayres and Russell also described sodium polyphosphates as potent synergists with several antibiotics (structures not shown).^327^ In the same study, citric acid (Figure 10) was also demonstrated to exhibit synergistic activity with erythromycin, novobiocin, rifampicin, methicillin, and gentamicin.^327^ In addition, 2,3-dimercaptosuccinic acid (Figure 10), clinically used in the treatment of lead intoxication, was also found to potentiate the activity of hydrophobic antibiotics.^323^ The synergistic activity of 2,3-dimercaptosuccinic acid was attributed to an OM-permeabilizing mechanism, as evidenced by increased NPN uptake in bacterial cells treated with the compound.^323^

## Concluding Remarks

New strategies are required to address the growing threat posed by MDR Gram-negative pathogens. To this end, a large and growing number of synergists capable of potentiating Gram-positive-specific antibiotics against Gram-negative bacteria have been described in the literature to date. Within this Review, we provide the reader with a comprehensive and up-to-date overview of those synergists reported to have a demonstrated OM-targeting mechanism. We also draw attention to the importance of selective OM disruption, a factor that has often been overlooked by researchers when characterizing their synergists. In this regard, and based on our assessment of the literature, the majority of hemolysis studies reported for such synergists use relatively short incubation times compared to the incubation times actually used in assessing synergy (i.e., in checkerboard assays). Based on our own experience, not only is the concentration at which hemolysis is assessed relevant, but incubation time can also make a significant difference in describing a compound as hemolytic or not. For example, in cases where 5% hemolysis is reported after 1 h, it is our experience that such compounds are often much more hemolytic after overnight incubation. For this reason, we have included both the concentrations and incubation times of the synergists described in this Review. Doing so provides for a more honest and accurate assessment of the OM specificity of these synergists.

To provide a means of comparing the relative activity of the synergists here summarized, we have emphasized their FICI values, a descriptor broadly applied as a scale to quantify synergistic potency. However, another important consideration that is not directly revealed by the FICI is, of course, the concentration at which a synergist actually potentiates the companion antibiotic. As for the concentrations of the antibiotics being potentiated, we suggest using the corresponding Gram-positive breakpoints as a guide for assessing whether the synergistic MICs determined against Gram-negative bacteria (for which Gram-positive antibiotics have no breakpoint) are within therapeutically relevant concentrations. Also related to this is the importance of the pharmacokinetic/pharmacodynamic (PK/PD) profile of the synergist and how well it matches that of the antibiotic it potentiates. Given that the vast majority of antibiotic synergists reported to date have only been characterized using cell-based *in vitro* and biochemical assays, we have not touched on this. Clearly, significant *in vivo* studies are needed to establish and optimize such parameters and will be essential to the (pre)clinical development of any such synergist. It is also notable that OM-perturbing synergists have been investigated as a means of enhancing the effect of other multi-drug cocktails, further underscoring the importance of such PK/PD considerations. Specifically, addition of the polymyxin-derived SPR741 has recently been studied as a means of enhancing the activity of β-lactam/β-lactamase inhibitor combinations such as piperacillin–tazobactam.^328^ Given the challenges associated with developing anti-Gram-negative agents and therapeutic strategies, the pursuit of antibiotic synergists is likely to remain an active field of research for the years to come.

## Abbreviations

ΔF: α,β-didehydrophenylalanine
alle: d-*allo*-isoleucine
AMP: antimicrobial peptide
BAM: β-barrel assembly machine
Bip: biphenylalanine
BPEI: branched polyethylenimine
BPI: bactericidal/permeability-increasing protein
C_6_: hexanoyl
C_8_: octanoyl
C_10_: decanoyl
Cbz: benzyloxycarbonyl
Cha: cyclohexylalanyl
Dab: 2,4-diaminobutyric acid
DAPB: deacylpolymyxin B
dUSCL: dilipid ultrashort cationic lipopeptide
dUSTBP: dilipid ultrashort tetrabasic peptidomimetic
EDTA: ethylenediaminetetraacetate
ESBL: extended spectrum β-lactamase
FICI: fractional inhibitory concentration index
HMP: hexametaphosphate
HTS: high-throughput screening
LPS: lipopolysaccharide
MAC: membrane attack complex
MDR: multi-drug-resistant
MIC: minimum inhibitory concentration
MOX: moxifloxacin
Nal: β-naphthylalanine
NEB: nebramine
NMP: 1-(1-naphthylmethyl)piperazine
NPN: *N*-phenylnaphthalen-1-amine
NR: no data reported
OAK: oligo-acyl-lysyl
OM: outer membrane
PAR: paroxetine
PEDES: PEptide DEscriptors from Sequence
PG: phosphatidylglycerol
PK/PD: pharmacokinetic/pharmacodynamic
PMB: polymyxin B
PMBH: polymyxin B heptapeptide
PMBN: polymyxin B nonapeptide
PMBO: polymyxin B octapeptide
SAR: structure–activity relationship
SM: squalamine mimic
TOB: tobramycin
TPP: tetraphenylphosphonium
TriA_1_: tridecaptin A_1_
UTBLP: ultrashort tetrabasic lipopeptide
WHO: World Health Organization
